# A Molecular “Thermometer” for Measuring Effective Non‐Local Exchange

**DOI:** 10.1002/jcc.70436

**Published:** 2026-06-23

**Authors:** Stefan Grimme, Marcel Müller, Thomas Froitzheim, Andreas Hansen

**Affiliations:** ^1^ Mulliken Center for Theoretical Chemistry, Clausius‐Institute for Physical and Theoretical Chemistry University of Bonn Bonn Germany; ^2^ Department of Chemistry University of Toronto Toronto Ontario Canada; ^3^ Vector Institute for Artificial Intelligence Toronto Ontario Canada

**Keywords:** density functional theory, electron delocalization, hydrocarbons, nonlocal exchange, thermochemistry

## Abstract

Non‐local exchange (NLX) is a key ingredient for accurate density functional calculations, but its effective strength is often hard to quantify beyond simple global hybrids. To this end, we introduce a molecular probe based on the isomerization of hexaethynylbenzene to *carbo*‐benzene, a reaction with exceptional sensitivity to exchange effects. Using the isomerization energy, we define a simple relative measure, $t_{X}$, that gauges the effective NLX of a method on a scale ranging from 0 for the local density approximation to 100 for Hartree‐Fock. Across a broad set of density functional approximations, $t_{X}$ closely tracks the formal Hartree‐Fock exchange content of global hybrids, while revealing that most common GGAs, meta‐GGAs, and global hybrids still provide too little effective exchange. Several range‐separated hybrid, double hybrid, and some local hybrid functionals perform much better and closely approach the near‐basis‐set‐limit coupled‐cluster reference value of $t_{X}=60$. The $t_{X}$ values of various semiempirical quantum mechanical and machine‐learned interatomic potential methods indicate that they empirically account for NLX effects to a varying degree. Thus, the proposed reaction serves as a compact thermochemical benchmark for assessing and developing methods that aim to reliably describe NLX.

## Graphical abstract

The isomerization of hexaethynylbenzene to *carbo*‐benzene acts as a sensitive “thermometer” for the effective treatment of non‐local exchange effects in density functional, semiempirical, and machine‐learning methods.

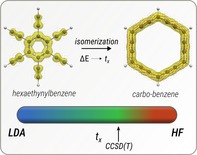

## 1 Introduction

Density functional theory (DFT), in its efficiently applicable Kohn‐Sham formulation, is the most widely used method in computational chemistry. The key ingredient in modern density functional approximations (DFAs) [1, 2] is the description of the exchange‐correlation (XC) energy. According to J. Perdew's metaphoric picture of Jacob's ladder (see Table 1) [3], DFAs are classified into rungs according to increasing accuracy starting from a purely local density approximation (LDA) [4, 5], to a generalized‐gradient approximation (GGA) on rung two [6], meta (m)‐GGA on rung three [7, 8], and hybrid [9] or double hybrid (DH) [10] on rungs four and five, respectively. Especially, the admixture of non‐local Fock‐exchange from Hartree‐Fock (HF) theory [11, 12] introduced by A. Becke in 1993 (see Equation 1) marked a milestone in the development of chemically accurate DFAs [13, 14]. So‐called hybrid functionals are among the most widely used DFAs, including functionals such as B3LYP or PBE0 [15, 16].

**TABLE 1:** Contribution of non‐local Fock exchange ($a_{X}$) in different types of DFAs according to “Jacob's ladder.” Here, r denotes a real‐space coordinate, $r_{12}$ is the inter‐electronic distance, and f is DFA‐specific function.

Rung on “Jacob's ladder”	$a_{X}$
Local (LDA)	0
Semi‐local GGA	0
Semi‐local meta‐GGA	0
Hybrid	> 0
Range‐separated hybrid	$f\left(r_{12}\right)$
Local hybrid	$f\left(r\right)$

Since there is a wide variety of approximations for the XC functional, comprehensive benchmark studies [17] and best practice guidelines [18] are indispensable. However, for an initial assessment of practical applicability, classification based on the admixture of non‐local Fock‐exchange is often already helpful. Overwhelming empirical evidence suggests that the treatment of non‐local exchange (NLX) effects is crucial for accurately describing thermochemistry and reaction barriers [17, 19, 20, 21], spectroscopic parameters [22, 23, 24, 25, 26, 27], and even equilibrium structures (e.g., bond‐length alternation [28, 29]).

Over the past few decades, many DFAs have been proposed that go beyond a simple linear mixing of local and NLX components in so‐called global hybrids (GHs).

(1) $$E_{X}=\left(1-a_{X}\right)\left(E_{X}^{\mathrm{LDA}/\left(m\right)\mathrm{GGA}}\right)+a_{X}E_{X}^{\text{Fock}}.$$

By parameterizing the mixing based on the inter‐electronic distance $r_{12}$, range‐separated hybrids (RSHs) [30] combine the correct long‐range exchange behavior of HF with beneficial semi‐local DFA exchange at short range. Local hybrids (LHs) [31] further increase flexibility by replacing the parameter $a_{X}$ in Equation (1) with a function of the real‐space electronic coordinate, tailoring the exchange admixture to the local electronic structure. In recent years, machine learning (ML) has also entered the field of DFA development [32, 33], frequently by targeting an optimal admixture of NLX in GHs and LHs [34, 35, 36, 37, 38]. The recent Skala mGGA [39] completely avoids admixture of Fock exchange in favor of a non‐local ML‐based XC functional, which may in the future warrant the addition of further intermediate rungs in Jacob's ladder. Nevertheless, it is often unclear how such functionals should be categorized and compared, given the lack of a single numerical value $a_{x}$ quantifying the extent of NLX effects.

**SCHEME 1:**
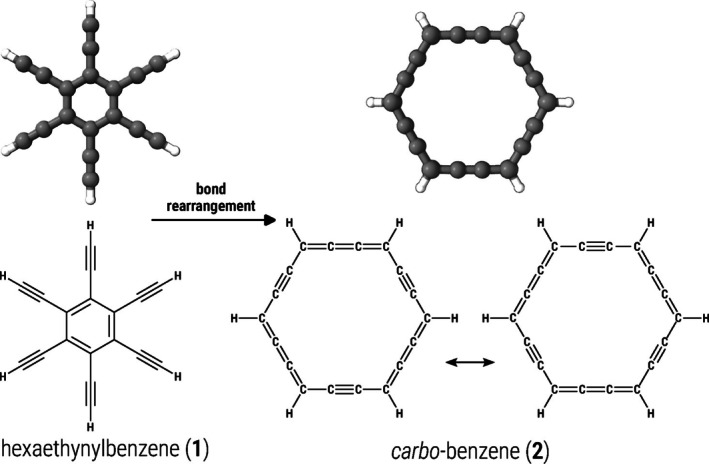
Bond rearrangement isomerization (BRI) of hexaethynylbenzene to *carbo*‐benzene. Top: Equilibrium geometries of both compounds on the RI‐MP2 [45, 46]/def2‐TZVPP [47, 48, 49] level of theory. Bottom: Corresponding Lewis structures.

In the course of current tight‐binding electronic structure developments [40], we stumbled over a seemingly simple hydrocarbon isomerization reaction of hexaethynylbenzene to so‐called *carbo*‐benzene [41, 42] that shows extreme sensitivity to NLX effects. Notably, the number of C‐C and C‐H bonds, as well as the number of sp (12) and sp^2^ (6) hybridized carbon atoms, remain unchanged during the reaction. We therefore refer to it as an (almost) homodesmotic bond rearrangement isomerization (BRI) [43, 44]. Strictly, the reaction is classified as isogyric due to the allene substructure in the resonance structures of *carbo*‐benzene (see Scheme 1). However, we prefer the nomenclature as almost homodesmotic based on the looser definition of equal numbers of sp^n^‐hybridized carbons without explicit reference to the bond order. The computed reaction energy $\Delta E=E\left(2\right)-E\left(1\right)$ for the isomerization of hexaethynylbenzene (1) to *carbo*‐benzene (2), as shown in Scheme 1, varies from about 66 kcal mol^−1^ at the HF level to −14 kcal mol^−1^ for LDA. The AO‐basis‐set‐converged coupled‐cluster (CC) reference value of 34.5 kcal mol^−1^ lies roughly in the middle of this range (vide infra). Normally, such huge effects are only observed in species with fractional charges and/or spin, for example, the prototypical H_2_^+^ dissociation [50, 51], making their occurrence in a reaction involving only neutral closed‐shell molecules particularly striking.

**FIGURE 1:**
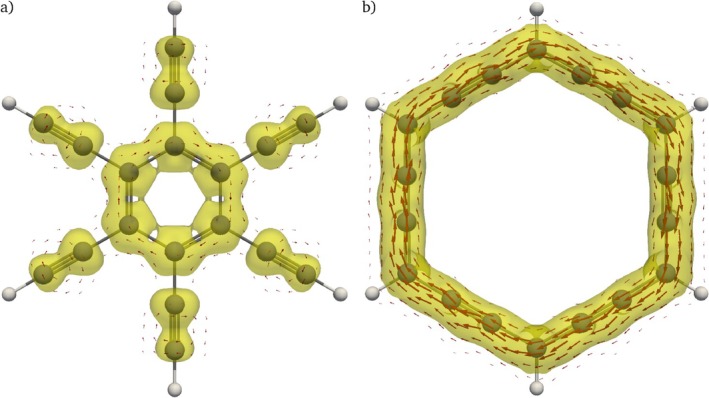
Combined scalar ACID (yellow, isovalue: 0.002) and vectorial MICD plot (red, selectively at 1 Bohr above the molecular plane vectors with a magnitude between 0.001 and 0.01 a.u., and scaled by a factor 5 for visibility) calculated at the PBE0 [16]/GIAO‐def2‐TZVPP [47, 48, 58] level of theory for hexaethynylbenzene (a) and *carbo*‐benzene (b).

Qualitatively, this observation can be rationalized by more pronounced π‐electron delocalization in (2) compared to (1), as indicated by the anisotropy of the current‐induced density (ACID) [52, 53, 54] and magnetically‐induced current density (MICD) [55, 56, 57] shown together in Figure 1. The integrated magnetically‐induced ring current increases more than fourfold for the BRI (from 8.1 nA T^−1^ in (1) to 34.1 nA T^−1^ in (2), see Supporting Information for details), which is in line with extensive previous studies on the aromaticity of (2) and various related compounds [59, 60, 61, 62, 63, 64]. Local or semi‐local DFAs generally overstabilize states with such delocalized electron densities, leading to the so‐called delocalization error [65, 66]. Analysis of the magnetically‐induced ring current and density‐corrected DFT calculations show that the resulting energetic error of semi‐local DFAs for the BRI is largely functional‐driven, that is, it arises from the limitations of the approximate energy functional rather than an excessively delocalized density (see Section S1 of the Supporting Information for details) [67].

Here, we propose the above reaction as a quantitative measure of NLX effects in thermochemistry. The idea is to provide a convenient test of the treatment of NLX effects in existing or newly proposed DFAs, even if $a_{X}$ is zero or given by a potentially complicated ML procedure. This approach also applies to commonly used RSHs and more recent LHs, for which the effective amount of exact exchange is often unclear, or even to semiempirical quantum mechanical (SQM) or machine‐learned interatomic potential (MLIP) methods without explicit (or only very approximate) account for NLX effects. Orbital energy gaps are often regarded as an alternative metric that focuses more on the XC potential than on the energy, and thus complements the thermochemical approach pursued here. While none of these approaches can replace comprehensive thermochemical benchmarking, they can provide valuable guidance in selecting a problem‐specific method or DFA.

## 2 Computational Details

The geometries of (1) and (2) were optimized at the RI‐MP2 [45, 46]/def2‐TZVPP [47, 48, 49] level of theory using the ORCA program package in version 6.0.1 [68]. All DFT calculations (except for the composite “‐3c” methods) consistently employ the def2‐TZVPP basis set and were mostly performed with ORCA using the libXC library version 7.0.0 [69] for the XC functionals. As an exception, LH calculations were performed with the Turbomole program version 8.0 [70], while selected DHs (ωB97M(2), PTPSS‐D3, and XYG3) were calculated with Q‐Chem version 6.3.1 [71]. The Skala and DM21 ML DFAs were used in PySCF version 2.11.0 [72, 73].

The xtb program version 6.7.1 [74] in combination with the tblite 0.4.0 library was used for GFN1‐xTB [75] and GFN2‐xTB [76] calculations, while g‐xTB calculations employ the standalone development program [40]. PM6‐D3H4X calculations were performed with MOPAC 2016 [77] via a wrapper in xtb 6.7.1. MLIPs were accessed via ASE [78] interface to the AIMNet2 [79] (version parametrized with ωB97M‐D3 reference values) and UMA‐sm [80] (version 1.0, with the task parameter set to omol) models.

Reference reaction energy values were computed with PNO‐LCCSD(T)‐F12b [81, 82, 83]/aug‐cc‐pV5Z [84, 85] (cc‐pV5Z for H; employing corresponding aug‐cc‐pV5Z+/JKFIT [86, 87] and aug‐cc‐pV5Z/MP2FIT [88] auxiliary basis sets) [83, 84, 85] with VTIGHT domain settings [89] and CABS singles correction using Molpro 2025.1 [90, 91]. The residual error for this highly accurate relative energy is estimated to be below 0.25 kcal mol^−1^ (see Supporting Information for details).

To investigate possible multi‐reference character of the electronic wavefunction, fractional occupation number weighted electron density (FOD) analysis [92, 93, 94] was performed at the PBE0‐D4/def2‐TZVPPD [95] level of theory with ORCA 6.0.1, employing an electronic temperature of $T_{\mathrm{el}}$ = 8830.5 K. Additionally, the first triplet excited states were calculated at the ωB97M‐V/def2‐TZVPPD UKS level of theory using the Turbomole program.

For the analysis of the delocalization and aromaticity in both systems, ACID [52, 53, 54] and MICD [55, 56, 57] were calculated at the PBE0/def2‐TZVPP level of theory using gauge‐including atomic orbitals (GIAOs) [58, 96] via the TURBOMOLE interface [97] to the GIMIC program version 2.2.1 [98]. Here, the magnetic field was applied perpendicular to the molecular plane, and the resulting MICD was integrated through one of the six half‐planes intersecting the carbon–carbon bonds of the original hexagonal benzene structure (see Supporting Information for details). ACID and MICD were plotted using ParaView version 5.13.2 [99].

## 3 Results and Discussion

The systems involved in the BRI are chemically simple, have high symmetry ($D_{6h}$) that enables fast calculations, and are insensitive to changes in the basis set and the optimized geometry used (see Supporting Information for details). Possible multi‐reference character of the electronic wavefunctions, especially for (2), has been investigated but found to be relatively mild: an analysis based on the FOD revealed only moderate amounts of “hot” electrons for (2) (slightly less than in, e.g., tetracene) and even less in (1) (see Supporting Information for details). This is further supported by relatively large first triplet excitation energies of 66.7 and 36.6 kcal mol^−1^ for (1) and (2), respectively. The multi‐reference diagnostics derived from the CCSD(T) calculations, such as T1, D1, and %TAE[(T)] [100], also show no signs for particularly difficult electronic structures. The corresponding values are well below the usual thresholds (T1 < 0.05, D1 < 0.150, %TAE[(T)] < 5%) [101] for both 1 (T1 = 0.013, D1 = 0.030, %TAE[(T)] = 2.84%) and 2 (T1 = 0.013, D1 = 0.032, %TAE[(T)] = 3.24%) and can therefore be clearly classified as single‐reference systems. We note, however, that with higher amounts of Fock exchange, the $D_{6h}$ symmetry reduces to $D_{3h}$ as the triple and double bonds localize and the mesomerization depicted in Scheme 1 breaks down. The effect of long‐range (London) dispersion interactions is relatively small (e.g., 10.1 kcal mol^−1^ with B3LYP‐D4 or 16.2 kcal mol^−1^ with HF‐D4). Thus, dispersion corrections are included for consistency in the following, but not further discussed (see Supporting Information for the actual dispersion contributions for the tested DFA).

The proposed relative (percentage) NLX measure $t_{X}$ is defined as

(2) $$t_{X}=100\cdot \frac{\Delta E-\Delta E_{\mathrm{LDA}}}{\Delta E_{\mathrm{HF}}-\Delta E_{\mathrm{LDA}}},$$

where ΔE is the computed reaction energy ($\Delta E=E\left(2\right)-E\left(1\right)$), such that $t_{X}=100$ for HF and $t_{X}=0$ for LDA. $t_{X}$ assumes a linear relation of the BRI reaction energy and NLX effects, motivated by previous observations that energetic quantities can show approximately linear sensitivities to the fraction of Fock exchange in hybrid density functionals [19, 102, 103]. However, we emphasize that $t_{X}$ represents an effective measure, which is conveniently mapped onto the extreme scale between LDA and HF and not a literal percentage of NLX. It should offer a helpful scale to compare a DFA's ability to capture NLX effects and inform the DFAs choice, given a problem‐specific NLX sensitivity. Since the CC reference reaction energy amounts to 34.5 kcal mol^−1^, the correct (CC‐based) effective NLX for this particular reaction is $t_{X}=60.0$. We note that defining $t_{X}$ via the reaction energy is not the only possible way to map the strong NLX dependence of the BRI into a relative measure. Quantities such as the gap between the highest‐occupied and lowest‐unoccupied molecular orbitals in 1 and 2 show a clear correlation with the $t_{X}$ value of a given method (see Section S7 of the Supporting Information for details), but remain restricted to molecular orbital‐based mean‐field methods. Table 2 collects both thermochemical data and the resulting $t_{X}$‐measure for a series of common DFAs from all rungs of Jacob's ladder (see Supporting Information for further examples).

**TABLE 2:** Formal amount of Fock exchange $a_{X}$ in %, reaction energies ΔE in kcal mol^−1^, and the NLX measure $t_{X}$ defined in Equation (2) for a selection DFAs and HF (see Supporting Information for further examples). The methods are ordered based on the $t_{X}$ measure. All DFT calculations employ the def2‐TZVPP basis set and a functional‐specific dispersion correction.

DFA	$a_{X}$	ΔE	$t_{X}$
LDA [4, 104]	0	−13.6	0.0
PBE‐D4 [9]	0	−10.7	3.6
M06L‐D4 [105]	0	−9.2	5.4
TPSS‐D4 [106]	0	−7.8	7.2
r^2^SCAN‐D4 [107]	0	−6.5	8.8
BLYP‐D4 [108, 109]	0	−6.2	9.2
B97M‐V [110]	0	−4.7	11.1
revPBE‐D4 [111]	0	−4.4	11.4
SCAN‐D4 [112]	0	−4.4	11.5
RPBE‐D4 [113]	0	−3.8	12.3
MN15‐L‐D3(0) [114]	0	−3.7	12.4
M11‐L‐D3(0) [115]	0	−3.3	12.9
TPSSh‐D4 [116]	10	−1.9	14.6
r^2^SCANh‐D4 [117]	10	−1.1	15.6
HSE06‐D4 [118]	25–0	2.7	20.3
B3LYP‐D4 [15]	20	4.5	22.6
PBE0‐D4 [16]	25	5.2	23.4
PW6B95‐D4 [119]	28	7.1	25.9
r^2^SCAN0‐D4 [117]	25	7.4	26.1
M06‐D4 [105]	27	10.1	29.5
PBE0‐DH [120]	50	12.6	32.7
TMHF‐D4 [121]	0–100	13.0	33.2
B2PLYP‐D4 [10]	53	13.2	33.5
PBE38‐D4 [17]	37.5	13.5	33.8
LH20t‐D4 [122]	0–100	14.3	34.8
SCS‐B2PLYP‐D4 [123]^a^	53	17.1	38.3
MN15‐D3(BJ) [124]	44	18.0	39.4
Pr^2^SCAN50‐D4 [125]	50	18.9	40.5
PWPB95‐D4 [126]	50	18.9	40.6
SOS‐B2PLYP‐D4 [123]^a^	53	19.1	40.8
CF22D [35]	46.3	19.2	41.0
PBE‐QIDH [127]	69	19.8	41.7
LH25nP‐D4 [38]	0–100	20.2	42.2
Skala‐D3 [39]	0	20.9	43.1
LH24n‐D4 [37]	0–100	21.1	43.3
r^2^SCAN50‐D4 [117]	50	21.8	44.2
CAM‐B3LYP‐D4 [128]	19–65	22.7	45.3
M06‐2X‐D3(0) [105]	54	23.4	46.1
BHHLYP‐D4 [13]	50	23.4	46.2
κPr^2^SCAN50‐D4 [125]	50	24.9	48.0
DM21‐D3(BJ) [34]	0–100	26.6	50.2
revDSD‐PBEP86‐D4 [129]	69	26.6	50.2
ωB97XD [130]	19.6–100	28.3	52.3
ωLH23tdE‐D4 [131]	0–100	31.8	56.7
ωr2SCAN‐D4 [125]	0–100	31.9	56.9
ωPr2SCAN50‐D4 [125]	50–100	32.6	57.7
LC‐BLYP‐D4 [132]	0–100	32.7	57.8
ωLH22t‐D4 [133]	0–100	33.4	58.6
ωLH25tdE‐D4 [134]	0–100	34.0	59.4
**CCSD(T) reference**	—	**34.5**	**60.0**
RSX‐QIDH [135]	69–100	35.1	60.8
M11‐D3(BJ) [136]	42.8–100	35.6	61.4
ωB97M(2) [137]	62.2	36.0	61.9
CAM‐QTP‐01‐D4 [138]	23–100	36.3	62.3
ωB97M‐V [139]	15–100	36.7	62.8
ωB97X‐V [140]	16.7–100	37.4	63.7
RSX‐0DH [141]	50–100	39.5	66.3
ωB97X‐D4 [142]	16.7–100	41.2	68.5
SCS‐ωB2PLYP‐D4 [123]^a^	53–100	41.5	68.8
LC‐PBE‐D4 [143]	0–100	49.8	79.2
HF‐D4 [11, 12]	100	66.5	100.0

^a^ DFT‐D4 parameters adapted from the canonical DH.

**FIGURE 2:**
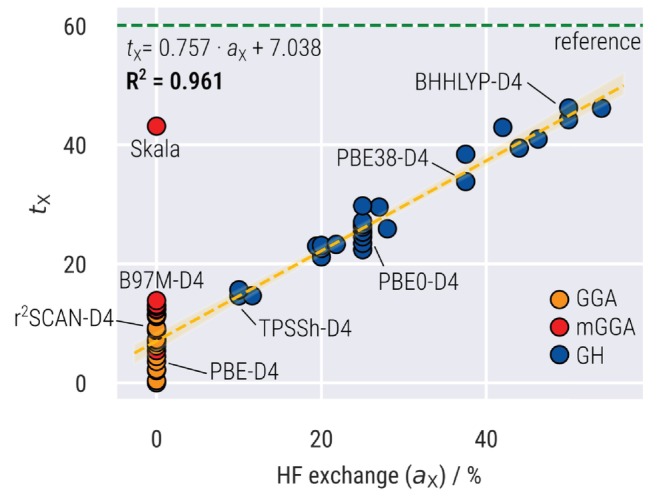
Proposed relative NLX measure $t_{X}$ (see Equation 2) versus the formal amount of exact Fock exchange ($a_{X}$) for a series of GGA (orange), mGGA (red), and GH (blue) DFAs (corresponding $t_{X}$ values in Table 2 and the Supporting Information). The dashed yellow line corresponds to a linear regression of $t_{X}$ with $a_{X}$ for the hybrid DFAs with a confidence interval of 95% (translucent band). The dashed horizontal green line corresponds to the expected $t_{x}$ for the reference reaction energy.

Figure 2 visualizes the proposed NLX measure, $t_{X}$, as a function of the formally admixed exact Fock exchange fraction, $a_{X}$. We focus initially on the subset of GHs (blue), where the non‐locality of the exchange effects is unambiguously quantified by $a_{X}$. Most notably, $t_{X}$ correlates closely with the $a_{X}$ fraction for the investigated GHs with a high Pearson correlation coefficient (PCC) of 0.961. Data points above the regression line indicate that the DFA intrinsically accounts for NLX effects through its semi‐local components. Linear regression maps the range from (semi‐)local DFAs ($a_{X}=0\%$) to HF ($a_{X}=100\%$) onto a slightly compressed $t_{X}$ range of roughly 7–83. This compression likely arises from the predominant use of semi‐local DFAs in the construction of GHs, which already slightly reduce the delocalization error of the purely local LDA used to define $t_{X}$ (vide infra). When comparing GHs based on different DFAs with the same $a_{X}$ fraction, we observe a slight scatter in the $t_{X}$ values (e.g., between 23.4 (PBE0‐D4) and 26.2 (r^2^SCAN0‐D4) at $a_{X}=25\%$). Such differences arise from NLX effects already present in the semi‐local components of the GH, which seem surprisingly small at only a few percent. Thus, even for modern third‐rung mGGA components, exact exchange admixture still plays the predominant role in counteracting delocalization error. However, none of the commonly employed GHs considered here admixes enough exact exchange to reach the reference value of $t_{X}=60.0$ (BHHLYP‐D4 with $a_{X}=50\%$ achieves the highest $t_{X}$ value for a GH at 46.2). Extrapolation of the linear $t_{X}$ versus $a_{X}$ trend for the tested global hybrids suggests that a conventional GH would require approximately 76.5% exact exchange to do so.

**FIGURE 3:**
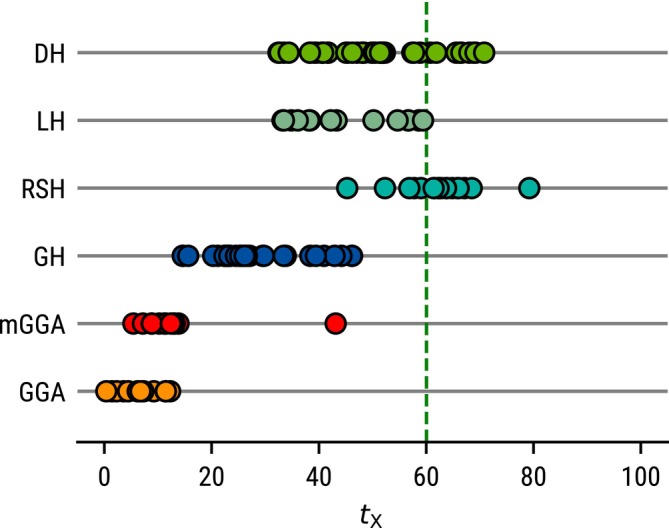
Proposed relative NLX measure $t_{X}$ (see Equation 2) for various functionals grouped as GGA (orange), mGGA (red), GH (blue), RSH (turquoise), LH (pale green), DH (green) DFAs. The dashed vertical green line corresponds to the expected $t_{x}$ needed to exactly match the CC reference reaction energy.

After validating the $t_{X}$ measure for GHs, we can now compare DFAs where the degree of non‐locality in the exchange treatment is less apparent. Figure 3 plots $t_{X}$ for the tested DFAs grouped by their respective functional classes. As in the case of GHs, all semi‐local (m)GGAs (orange and red) underestimate NLX effects, yielding mostly $t_{X}$ values below 20. While third‐rung mGGAs have, on average, a slightly higher $t_{X}$ than rung‐two GGAs, the differences are negligible compared to the remaining error. The ML‐based Skala functional represents the only striking exception: although $a_{X}$ is formally zero, its effective $t_{X}$ value of 43.1 is comparable to GHs with large fractions of Fock exchange. This suggests that the non‐local construction of the Skala XC functional effectively emulates the role of exact exchange in hybrids, even though it is still not quite sufficient for the present example.

According to the introduced “thermometer” for NLX, DFAs require $t_{X}$ values between 55 and 65 to reproduce the CC reference reaction energy with reasonable accuracy (10% or ±3.5 kcal mol^−1^). Among all tested DFAs, most RSHs and several DHs fall into this region, whereas only a few LHs do so. CAM‐B3LYP‐D4 ($t_{X}=45.2$) and LC‐PBE‐D4 ($t_{X}=79.2$) are notable exceptions among the RSHs, illustrating two distinct issues. The former admixes Fock exchange only up to 65% at large inter‐electronic distances, rather than the full 100% expected from Hartree‐Fock theory [128], whereas the latter includes too much Fock exchange already at short range due to its unusually large range‐separation parameter of 0.47 Bohr^−1^ [143]. In particular, the long‐range admixture of 100% Fock exchange appears to be crucial, as is also evident from the tested LHs and DHs. While all tested range‐separated LHs fall within the narrow $t_{X}$ range between 55 and 60, a purely local modification of the Fock exchange admixture appears insufficient (e.g., LH20t‐D4 with $t_{X}=34.8$). Even ML‐based local mixing functions, such as those used in LH25nP‐D4 ($t_{X}=42.2$) and DM21‐D3(BJ) ($t_{X}=50.2$), cannot fully eliminate the delocalization error. For DHs, a similar picture emerges, with all range‐separated DHs having $t_{X}$ values between 55 and 69, while global DHs yield lower values (e.g., B2PLYP with $t_{X}=33.5$ and revDSD‐PBEP86‐D4 with $t_{X}=50.2$). Taken together, these results suggest that the $t_{X}$ measure is predominantly determined by long‐range NLX effects and is much less sensitive to other details of a functional, such as the treatment of correlation.

To assess whether the $t_{X}$ measure extends meaningfully beyond systems characterized by strong π‐electron delocalization, we compare $t_{X}$ with the mean deviations (MDs) for the BH76 barrier‐height benchmark set [17, 144]. This benchmark comprises transition states of small‐molecule reactions, including radicals and charged species. For selected functionals, the MDs for BH76 and $t_{x}$ show moderate correlation, with $R^{2}=0.573$ (see Section S6 of the Supporting Information for detailed results). This correlation is mainly weakened by a small number of well‐performing (m)GGAs functionals, most notably BLYP and the Minnesota functionals MN15‐L, M11‐L, and M06‐L. Excluding these functionals increases the correlation to $R^{2}=0.858$, suggesting that $t_{X}$ can serve as a useful qualitative indicator for missing NLX contributions in the description of transition states.

Finally, we extend the use of the NLX measure beyond the comparison of DFAs within a fixed basis set. The composite DFT (“‐3c”), SQM, and MLIP methods in Table 3 span a remarkably wide range of effective NLX performance, revealing both the promise and limitations of computationally efficient approaches. Among the composite DFT methods, the results follow the expected trend with the rungs of Jacob's ladder. B97‐3c, lacking any Fock exchange, yields a strongly negative reaction energy and a near‐zero $t_{X}$ of 1.4, essentially behaving like a standard GGA. r^2^SCAN‐3c improves only modestly to $t_{X}$ = 10.2, consistent with its mGGA character. PBEh‐3c, with 42% formal Fock exchange, reaches $t_{X}$ = 33.4, placing it within the range of GHs but still well below the target of 60.0. Among the composite methods, the range‐separated ωB97X‐3c performs best with $t_{X}$ = 64.8, thereby slightly overshooting the CC reference. The pathological behavior of HF‐3c ($t_{X}$ = 138.5) reflects the well‐known overcorrection of pure HF for delocalized systems, combined with limitations due to the minimal basis set used.

**TABLE 3:** Formal amount of Fock exchange $a_{X}$ in %, reaction energies ΔE in kcal mol^−1^, and the NLX measure $t_{X}$ for a set of composite DFT, SQM, and MLIP methods.

DFA/SQM/MLIP	$a_{X}$	ΔE	$t_{X}$
B97‐3c [145]	0	−12.5	1.4
r^2^SCAN‐3c [146]	0	−5.4	10.2
PBEh‐3c [147]	42	13.2	33.4
ωB97X‐3c [148]	16.7–100	38.3	64.8
HF‐3c [149]	100	97.3	138.5
GFN1‐xTB [75]	0	14.0	34.5
GFN2‐xTB [76]	0	9.8	29.2
g‐xTB [40]	15–100	36.7	62.7
PM6‐D3H4X [150, 151]	100	44.0	72.0
AIMNet2 [79]	—	6.7	25.3
UMA‐sm [80]	—	37.4	63.7

The tested tight‐binding (TB) methods show a clear progression with increasing sophistication. GFN1‐xTB and GFN2‐xTB, both formally without Fock exchange, yield $t_{X}$ values of 34.5 and 29.2, respectively, already substantially above the GGA baseline. This suggests that their semiempirical parameterization implicitly captures some NLX‐like effects, as expected from the use of higher‐level reference data during their fitting. Most notably, g‐xTB achieves a $t_{X}$ value of 62.7, approaching the CC target region impressively for an SQM method. Given that g‐xTB employs a range‐dependent approximate exchange treatment, this result is encouraging and suggests that the method captures a meaningful fraction of the physical NLX effects, even if the delocalization error is not fully corrected. PM6‐D3H4X, with formally 100% HF exchange in its semiempirical framework, overshoots with $t_{X}$ = 72.0, although not to the same extent as HF itself.

The MLIP results are striking and illustrative of the current state of the field. UMA‐sm achieves a $t_{X}$ value of 63.7, essentially matching the CC reference, which is remarkable for a method with no explicit quantum mechanical treatment of exchange. This implies that the training data and architecture, which is aimed at universality, have led to an implicit encoding of NLX‐like effects. In contrast, AIMNet2 yields a clearly incorrect reaction energy, suggesting that the electronic reorganization in this BRI is not captured accurately. Since both UMA‐sm and AIMNet2 aim to reproduce RSH‐DFT reference data, it appears reasonable to assume that such significant changes in electron delocalization are better represented in the larger OMol25 data set [152] used for UMA‐sm.

## 4 Conclusion

We presented a simple molecular “thermometer” that quantifies effective non‐local exchange (NLX) via the isomerization energy of hexaethynylbenzene to *carbo*‐benzene. The newly introduced relative $t_{X}$ measure quantifies NLX effects on a scale ranging from 0 for local density approximation to 100 for Hartree‐Fock and requires only two comparably inexpensive energy evaluations. This thermochemistry‐based approach provides a practical complement to orbital‐gap or potential‐based diagnostics. Moreover, it is based on electronically relatively simple hydrocarbons, thereby avoiding interference with charge transfer or static correlation effects.

To test the $t_{X}$ measure, we computed a high‐level coupled‐cluster reference value for the “thermometer” reaction and compared results across a comprehensive set of DFAs, SQM methods, and MLIPs. $t_{X}$ closely correlates with the formal Fock exchange admixture in global hybrids (GHs), and we showed that the effective NLX in commonly used GGAs, mGGAs, and GHs is insufficient to describe the investigated reaction accurately. Meanwhile, range‐separated hybrids, as well as their local hybrid and double hybrid variants, closely approach the reference $t_{X}$ value of 60. In particular, the full treatment of long‐range NLX effects via the admixture of 100% long‐range Fock exchange is crucial for removing the delocalization error dominant in this isomerization. These results imply that the introduced molecular “thermometer” may serve as a practical initial probe for choosing and developing DFAs, SQM methods, and MLIPs that accurately capture NLX in chemically relevant problems.

## Conflicts of Interest

The authors declare no conflicts of interest.

## Supporting information


**Data S1:** jcc70436‐sup‐0001‐Supinfo.pdf.

**Data S2:** jcc70436‐sup‐0002‐Supinfo.zip

## Data Availability

The data that supports the findings of this study are available in the Supporting Information of this article.

## References

[1] P. Hohenberg and W. Kohn , “Inhomogeneous Electron Gas,” Physical Review 136 (1964): B864–B871.

[2] W. Kohn and L. J. Sham , “Self‐Consistent Equations Including Exchange and Correlation Effects,” Physical Review 140 (1965): A1133–A1138.

[3] J. P. Perdew and K. Schmidt , “AIP Conference Proceedings,” (2001), 577, 1.

[4] P. a. M. Dirac , “Note on Exchange Phenomena in the Thomas Atom,” Mathematical Proceedings of the Cambridge Philosophical Society 26 (1930): 376–385.

[5] J. C. Slater , “A Simplification of the Hartree‐Fock Method,” Physical Review 81 (1951): 385–390.

[6] J. P. Perdew and W. Yue , “Accurate and Simple Density Functional for the Electronic Exchange Energy: Generalized Gradient Approximation,” Physical Review B 33 (1986): 8800–8802. 10.1103/physrevb.33.8800 9938293

[7] T. Van Voorhis and G. E. Scuseria , “A Novel Form for the Exchange‐Correlation Energy Functional,” Journal of Chemical Physics 109 (1998): 400–410.

[8] J. P. Perdew , S. Kurth , A. Zupan , and P. Blaha , “Accurate Density Functional With Correct Formal Properties: A Step Beyond the Generalized Gradient Approximation,” Physical Review Letters 82 (1999): 2544–2547.

[9] J. P. Perdew , M. Ernzerhof , and K. Burke , “Rationale for Mixing Exact Exchange With Density Functional Approximations,” Journal of Chemical Physics 105 (1996): 9982–9985.

[10] S. Grimme , “Semiempirical Hybrid Density Functional With Perturbative Second‐Order Correlation,” Journal of Chemical Physics 124 (2006): 124. 10.1063/1.2148954 16438568

[11] D. R. Hartree , “The Wave Mechanics of an Atom With a Non‐Coulomb Central Field. Part II. Some Results and Discussion,” Mathematical Proceedings of the Cambridge Philosophical Society 24 (1928): 111–132.

[12] V. Fock , “Näherungsmethode Zur Lösung Des Quantenmechanischen Mehrkörperproblems,” Zeitschrift für Physik 61 (1930): 126–148.

[13] A. D. Becke , “A New Mixing of Hartree–Fock and Local Density‐Functional Theories,” Journal of Chemical Physics 98 (1993): 1372–1377.

[14] A. D. Becke , “Density‐Functional Thermochemistry. III. The Role of Exact Exchange,” Journal of Chemical Physics 98 (1993): 5648–5652.

[15] P. J. Stephens , F. J. Devlin , C. F. Chabalowski , and M. J. Frisch , “Ab Initio Calculation of Vibrational Absorption and Circular Dichroism Spectra Using Density Functional Force Fields,” Journal of Physical Chemistry 98 (1994): 11623–11627.

[16] C. Adamo and V. Barone , “Toward Reliable Density Functional Methods Without Adjustable Parameters: The PBE0 Model,” Journal of Chemical Physics 110 (1999): 6158–6170.

[17] L. Goerigk , A. Hansen , C. Bauer , S. Ehrlich , A. Najibi , and S. Grimme , “A Look at the Density Functional Theory Zoo With the Advanced GMTKN55 Database for General Main Group Thermochemistry, Kinetics and Noncovalent Interactions,” Physical Chemistry Chemical Physics 19 (2017): 32184–32215. 29110012 10.1039/c7cp04913g

[18] M. Bursch , J.‐M. Mewes , A. Hansen , and S. Grimme , “Best‐Practice DFT Protocols for Basic Molecular Computational Chemistry,” Angewandte Chemie 134 (2022): e202205735. 10.1002/anie.202205735 PMC9826355 36103607

[19] V. Vennelakanti , A. Nandy , and H. J. Kulik , “The Effect of Hartree‐Fock Exchange on Scaling Relations and Reaction Energetics for C–H Activation Catalysts,” Topics in Catalysis 65 (2022): 296–311.

[20] J. Liang and M. Head‐Gordon , “Gold‐Standard Chemical Database 137 (GSCDB137): A Diverse Set of Accurate Energy Differences for Assessing and Developing Density Functionals,” Journal of Chemical Theory and Computation 21 (2025): 12601–12621. 41325623 10.1021/acs.jctc.5c01380 PMC12746454

[21] Y. Pillai , H. G. A. Burton , and D. J. Wales , “Effect of Exact Exchange on the Energy Landscape in Self‐Consistent Field Theory,” Journal of Chemical Theory and Computation 21 (2025): 1203–1212. 39824763 10.1021/acs.jctc.4c01404 PMC11823404

[22] J. C. Howard , J. D. Enyard , and G. S. Tschumper , “Assessing the Accuracy of Some Popular DFT Methods for Computing Harmonic Vibrational Frequencies of Water Clusters,” Journal of Chemical Physics 143 (2015): 214103. 26646865 10.1063/1.4936654

[23] N. Palanisamy and S. Banik , “Exploring the Accuracy of Density Functionals for Anharmonic Vibrations of Polycyclic Aromatic Hydrocarbons,” Journal of Physical Chemistry A 129 (2025): 7794–7807. 40801825 10.1021/acs.jpca.5c03333

[24] M. Dierksen and S. Grimme , “The Vibronic Structure of Electronic Absorption Spectra of Large Molecules: A Time‐Dependent Density Functional Study on the Influence of “Exact” Hartree−Fock Exchange,” Journal of Physical Chemistry A 108 (2004): 10225–10237.

[25] J. Shee and M. Head‐Gordon , “Predicting Excitation Energies of Twisted Intramolecular Charge‐Transfer States With the Time‐Dependent Density Functional Theory: Comparison With Experimental Measurements in the Gas Phase and Solvents Ranging From Hexanes to Acetonitrile,” Journal of Chemical Theory and Computation 16 (2020): 6244–6255. 32816472 10.1021/acs.jctc.0c00635

[26] T. Froitzheim , S. Grimme , and J.‐M. Mewes , “Either Accurate Singlet–Triplet Gaps or Excited‐State Structures: Testing and Understanding the Performance of TD‐DFT for TADF Emitters,” Journal of Chemical Theory and Computation 18 (2022): 7702–7713. 36409831 10.1021/acs.jctc.2c00905

[27] T. Gasevic , J. B. Kleine Büning , S. Grimme , and M. Bursch , “Benchmark Study on the Calculation of207Pb NMR Chemical Shifts,” Inorganic Chemistry 63 (2024): 5052–5064. 38446045 10.1021/acs.inorgchem.3c04539 PMC10951955

[28] D. Jacquemin and C. Adamo , “Bond Length Alternation of Conjugated Oligomers: Wave Function and DFT Benchmarks,” Journal of Chemical Theory and Computation 7 (2011): 369–376. 26596158 10.1021/ct1006532

[29] T. Körzdörfer , R. M. Parrish , J. S. Sears , C. D. Sherrill , and J.‐L. Brédas , “On the Relationship Between Bond‐Length Alternation and Many‐Electron Self‐Interaction Error,” Journal of Chemical Physics 137 (2012): 124305. 23020329 10.1063/1.4752431

[30] A. Savin and H.‐J. Flad , “Density Functionals for the Yukawa Electron‐Electron Interaction,” International Journal of Quantum Chemistry 56 (1995): 327–332.

[31] J. Jaramillo , G. E. Scuseria , and M. Ernzerhof , “Local Hybrid Functionals,” Journal of Chemical Physics 118 (2003): 1068–1073.

[32] K. Bystrom and B. Kozinsky , “Nonlocal Machine‐Learned Exchange Functional for Molecules and Solids,” Physical Review B 110 (2024): 075130.

[33] R. Akashi , M. Sogal , and K. Burke , “Can Machines Learn Density Functionals? Past, Present, and Future of ML in DFT,” (2025), arXiv:2503.01709 [physics].

[34] J. Kirkpatrick , B. McMorrow , D. H. P. Turban , et al., “Pushing the Frontiers of Density Functionals by Solving the Fractional Electron Problem,” Science 374 (2021): 1385–1389. 34882476 10.1126/science.abj6511

[35] Y. Liu , C. Zhang , Z. Liu , D. G. Truhlar , Y. Wang , and X. He , “Supervised Learning of a Chemistry Functional With Damped Dispersion,” Nature Computational Science 3 (2023): 48–58. 38177952 10.1038/s43588-022-00371-5 PMC10766516

[36] D. Khan , A. J. A. Price , B. Huang , M. L. Ach , and O. A. von Lilienfeld , “Adapting Hybrid Density Functionals With Machine Learning,” Science Advances 11 (2025): eadt7769. 39888985 10.1126/sciadv.adt7769 PMC11784814

[37] A. Wodyński , K. Glodny , and M. Kaupp , “Data‐Driven Improvement of Local Hybrid Functionals: Neural‐Network‐Based Local Mixing Functions and Power‐Series Correlation Functionals,” Journal of Chemical Theory and Computation 21 (2025): 762–775. 39805000 10.1021/acs.jctc.4c01503 PMC11780747

[38] A. Wodyński and M. Kaupp , “Local‐Hybrid Functional With a Composite Local Mixing Function Built From a Neural Network and a Strong‐Correlation Model,” Journal of Computational Chemistry 47 (2026): e70294. 41579855 10.1002/jcc.70294 PMC12831636

[39] G. Luise , C.‐W. Huang , T. Vogels , et al., “Accurate and Scalable Exchange‐Correlation With Deep Learning,” (2025), arXiv:2506.14665 [physics] version: 1.

[40] T. Froitzheim , M. Müller , A. Hansen , and S. Grimme , “g‐xTB: A General‐Purpose Extended Tight‐Binding Electronic Structure Method for the Elements H to Lr (Z = 1–103),” (2025).

[41] R. Chauvin , ““Carbomers”. I. A General Concept of Expanded Molecules,” Tetrahedron Letters 36 (1995): 397–400.

[42] R. Chauvin , ““Carbomers”. II. En Route to [C,C]6carbo‐Benzene,” Tetrahedron Letters 36 (1995): 401–404.

[43] P. George , M. Trachtman , C. W. Bock , and A. M. Brett , “An Alternative Approach to the Problem of Assessing Stabilization Energies in Cyclic Conjugated Hydrocarbons,” Theoretica Chimica Acta 38 (1975): 121–129.

[44] S. E. Wheeler , K. N. Houk , P. v. R. Schleyer , and W. D. Allen , “A Hierarchy of Homodesmotic Reactions for Thermochemistry,” Journal of the American Chemical Society 131 (2009): 2547–2560. 19182999 10.1021/ja805843n PMC2711007

[45] C. Møller and M. S. Plesset , “Note on an Approximation Treatment for Many‐Electron Systems,” Physics Review 46 (1934): 618–622.

[46] M. Feyereisen , G. Fitzgerald , and A. Komornicki , “Use of Approximate Integrals in Ab Initio Theory. An Application in MP2 Energy Calculations,” Chemical Physics Letters 208 (1993): 359–363.

[47] F. Weigend and R. Ahlrichs , “Balanced Basis Sets of Split Valence, Triple Zeta Valence and Quadruple Zeta Valence Quality for H to Rn: Design and Assessment of Accuracy,” Physical Chemistry Chemical Physics 7 (2005): 3297–3305. 16240044 10.1039/b508541a

[48] F. Weigend , “Accurate Coulomb‐Fitting Basis Sets for H to Rn,” Physical Chemistry Chemical Physics 8 (2006): 1057–1065. 16633586 10.1039/b515623h

[49] A. Hellweg , C. Hättig , S. Höfener , and W. Klopper , “Optimized Accurate Auxiliary Basis Sets for RI‐MP2 and RI‐CC2 Calculations for the Atoms Rb to Rn,” Theoretical Chemistry Accounts 117 (2007): 587–597.

[50] R. Merkle , A. Savin , and H. Preuss , “Singly Ionized First‐Row Dimers and Hydrides Calculated With the Fully‐Numerical Density‐Functional Programnumol,” Journal of Chemical Physics 97 (1992): 9216–9221.

[51] D. R. Lonsdale and L. Goerigk , “The One‐Electron Self‐Interaction Error in 74 Density Functional Approximations: A Case Study on Hydrogenic Mono‐ and Dinuclear Systems,” Physical Chemistry Chemical Physics 22 (2020): 15805–15830. 32458849 10.1039/d0cp01275k

[52] R. Herges and D. Geuenich , “Delocalization of Electrons in Molecules,” Journal of Physical Chemistry A 105 (2001): 3214–3220.

[53] D. Geuenich , K. Hess , F. Köhler , and R. Herges , “Anisotropy of the Induced Current Density (ACID), a General Method to Quantify and Visualize Electronic Delocalization,” Chemical Reviews 105 (2005): 3758–3772. 16218566 10.1021/cr0300901

[54] H. Fliegl , J. Jusélius , and D. Sundholm , “Gauge‐Origin Independent Calculations of the Anisotropy of the Magnetically Induced Current Densities,” Journal of Physical Chemistry A 120 (2016): 5658–5664. 27322880 10.1021/acs.jpca.6b03950

[55] J. Jusélius , D. Sundholm , and J. Gauss , “Calculation of Current Densities Using Gauge‐Including Atomic Orbitals,” Journal of Chemical Physics 121 (2004): 3952–3963. 15332941 10.1063/1.1773136

[56] D. Sundholm , H. Fliegl , and R. J. Berger , “Calculations of Magnetically Induced Current Densities: Theory and Applications,” WIREs Computational Molecular Science 6 (2016): 639–678.

[57] H. Fliegl , S. Taubert , O. Lehtonen , and D. Sundholm , “The Gauge Including Magnetically Induced Current Method,” Physical Chemistry Chemical Physics 13 (2011): 20500. 21909556 10.1039/c1cp21812c

[58] F. London , “Théorie Quantique Des Courants Interatomiques Dans Les Combinaisons Aromatiques,” Journal de Physique et le Radium 8 (1937): 397–409.

[59] C. Lepetit , C. Godard , and R. Chauvin , “Aromaticity and Homoaromaticity of Annulene Ring Carbomers,” New Journal of Chemistry 25 (2001): 572–580.

[60] C. Lepetit , B. Silvi , and R. Chauvin , “ELF Analysis of Out‐Of‐Plane Aromaticity and In‐Plane Homoaromaticity in Carbo[N]Annulenes and [N]Pericyclynes,” Journal of Physical Chemistry A 107 (2003): 464–473.

[61] C. Zou , C. Lepetit , Y. Coppel , and R. Chauvin , “Ring Carbo‐Mers: From Questionable Homoaromaticity to Bench Aromaticity,” Pure and Applied Chemistry 78 (2006): 791–811.

[62] S. Jalife , M. Audiffred , R. Islas , et al., “The Inorganic Analogues of Carbo‐Benzene,” Chemical Physics Letters 610‐611 (2014): 209–212.

[63] K. Cocq , C. Lepetit , V. Maraval , and R. Chauvin , ““Carbo‐Aromaticity” and Novel Carbo‐Aromatic Compounds,” Chemical Society Reviews 44 (2015): 6535–6559. 26077437 10.1039/c5cs00244c

[64] D. Arias‐Olivares , A. Becerra‐Buitrago , L. C. García‐Sánchez , D. V. Moreno , and R. Islas , “In Silico Analysis of the Aromaticity of SomeCarbo‐Metallabenzenes andCarbo‐Dimetallabenzenes (Carbo‐Mers Proposed From Metallabenzenes),” ACS Omega 9 (2024): 10913–10928. 38463261 10.1021/acsomega.3c10049 PMC10918654

[65] P. Mori‐Sánchez , A. J. Cohen , and W. Yang , “Localization and Delocalization Errors in Density Functional Theory and Implications for Band‐Gap Prediction,” Physical Review Letters 100 (2008): 146401. 18518055 10.1103/PhysRevLett.100.146401

[66] K. R. Bryenton , A. A. Adeleke , S. G. Dale , and E. R. Johnson , “Delocalization Error: The Greatest Outstanding Challenge in Density‐Functional Theory,” WIREs Computational Molecular Science 13 (2023): e1631.

[67] M.‐C. Kim , E. Sim , and K. Burke , “Understanding and Reducing Errors in Density Functional Calculations,” Physical Review Letters 111 (2013): 073003. 23992062 10.1103/PhysRevLett.111.073003

[68] F. Neese , “Software Update: TheORCAProgram System—Version 6.0,” WIREs Computational Molecular Science 15 (2025): e70019.

[69] S. Lehtola , C. Steigemann , M. J. T. Oliveira , and M. A. L. Marques , “Recent Developments in Libxc — A Comprehensive Library of Functionals for Density Functional Theory,” SoftwareX 7 (2018): 1–5.

[70] Y. J. Franzke , C. Holzer , J. H. Andersen , et al., “TURBOMOLE: Today and Tomorrow,” Journal of Chemical Theory and Computation 19 (2023): 6859–6890. 37382508 10.1021/acs.jctc.3c00347 PMC10601488

[71] E. Epifanovsky , A. T. Gilbert , X. Feng , et al., “Software for the Frontiers of Quantum Chemistry: An Overview of Developments in the Q‐Chem 5 Package,” Journal of Chemical Physics 155 (2021): 084801. 34470363 10.1063/5.0055522 PMC9984241

[72] Q. Sun , X. Zhang , S. Banerjee , et al., “Recent Developments in the PySCF Program Package,” Journal of Chemical Physics 153 (2020): 024109. 32668948 10.1063/5.0006074

[73] A. Petrone , D. B. Williams‐Young , S. Sun , T. F. Stetina , and X. Li , “An Efficient Implementation of Two‐Component Relativistic Density Functional Theory With Torque‐Free Auxiliary Variables,” European Physical Journal B 91 (2018): 169.

[74] C. Bannwarth , E. Caldeweyher , S. Ehlert , et al., “Extended Tight‐Binding Quantum Chemistry Methods,” WIREs Computational Molecular Science 11 (2021): e1493.

[75] S. Grimme , C. Bannwarth , and P. Shushkov , “Robust and Accurate Tight‐Binding Quantum Chemical Method for Structures, Vibrational Frequencies, and Noncovalent Interactions of Large Molecular Systems,” Journal of Chemical Theory and Computation 13 (2017): 1989–2009. 28418654 10.1021/acs.jctc.7b00118

[76] C. Bannwarth , S. Ehlert , and S. Grimme , “GFN2‐xTB—An Accurate and Broadly Parametrized Self‐Consistent Tight‐Binding Quantum Chemical Method With Multipole Electrostatics and Density‐Dependent Dispersion Contributions,” Journal of Chemical Theory and Computation 15 (2019): 1652–1671. 30741547 10.1021/acs.jctc.8b01176

[77] J. J. P. Stewart , “MOPAC2016,” (2016).

[78] A. Hjorth Larsen , J. Jørgen Mortensen , J. Blomqvist , et al., “The Atomic Simulation Environment—A Python Library for Working With Atoms,” Journal of Physics: Condensed Matter 29 (2017): 273002. 28323250 10.1088/1361-648X/aa680e

[79] D. M. Anstine , R. Zubatyuk , and O. Isayev , “AIMNet2: A Neural Network Potential to Meet Your Neutral, Charged, Organic, and Elemental‐Organic Needs,” Chemical Science 16 (2025): 10228–10244. 40342914 10.1039/d4sc08572h PMC12057637

[80] B. M. Wood , M. Dzamba , X. Fu , et al., “UMA: A Family of Universal Models for Atoms,” (2025), arXiv:2506.23971 [cs].

[81] Q. Ma and H.‐J. Werner , “Explicitly Correlated Local Coupled‐Cluster Methods Using Pair Natural Orbitals,” WIREs Computational Molecular Science 8 (2018): e1371.

[82] Q. Ma and H.‐J. Werner , “Scalable Electron Correlation Methods. 5. Parallel Perturbative Triples Correction for Explicitly Correlated Local Coupled Cluster With Pair Natural Orbitals,” Journal of Chemical Theory and Computation 14 (2018): 198–215. 29211961 10.1021/acs.jctc.7b01141

[83] H.‐J. Werner and A. Hansen , “Accurate Calculation of Isomerization and Conformational Energies of Larger Molecules Using Explicitly Correlated Local Coupled Cluster Methods in Molpro and ORCA,” Journal of Chemical Theory and Computation 19 (2023): 7007–7030. 37486154 10.1021/acs.jctc.3c00270

[84] T. H. J. Dunning , “Gaussian Basis Sets for Use in Correlated Molecular Calculations. I. The Atoms Boron Through Neon and Hydrogen,” Journal of Chemical Physics 90 (1989): 1007–1023.

[85] R. Kendall , T. H. J. Dunning , and R. J. Harrison , “Electron Affinities of the First‐Row Atoms Revisited. Systematic Basis Sets and Wave Functions,” Journal of Chemical Physics 96 (1992): 2257–6806.

[86] F. Weigend , “A Fully Direct RI‐HF Algorithm: Implementation, Optimised Auxiliary Basis Sets, Demonstration of Accuracy and Efficiency,” Physical Chemistry Chemical Physics 4 (2002): 4285–4291.

[87] G. Knizia and H.‐J. Werner , “Explicitly Correlated RMP2 for High‐Spin Open‐Shell Reference States,” Journal of Chemical Physics 128 (2008): 154103. 18433186 10.1063/1.2889388

[88] F. Weigend , A. Köhn , and C. Hättig , “Efficient Use of the Correlation Consistent Basis Sets in Resolution of the Identity MP2 Calculations,” Journal of Chemical Physics 116 (2002): 3175–3183.

[89] A. Hansen , P. J. Knowles , and H.‐J. Werner , “Accurate Calculation of Noncovalent Interactions Using PNO‐LCCSD(T)‐F12 in Molpro,” Journal of Physical Chemistry A 129 (2025): 4812–4833. 40388241 10.1021/acs.jpca.5c02316

[90] H.‐J. Werner , P. J. Knowles , F. R. Manby , et al., “The Molpro Quantum Chemistry Package,” Journal of Chemical Physics 152 (2020): 144107. 32295355 10.1063/5.0005081

[91] H.‐J. Werner , P. J. Knowles , P. Celani , et al., “MOLPRO, Version 2025.1, a Package of ab Initio Programs,” (2025), Stuttgart, Germany.

[92] S. Grimme and A. Hansen , “A Practicable Real‐Space Measure and Visualization of Static Electron‐Correlation Effects,” Angewandte Chemie International Edition 54 (2015): 12308–12313. 25882895 10.1002/anie.201501887

[93] C. A. Bauer , A. Hansen , and S. Grimme , “The Fractional Occupation Number Weighted Density as a Versatile Analysis Tool for Molecules With a Complicated Electronic Structure,” Chemistry—A European Journal 23 (2017): 6150. 27906486 10.1002/chem.201604682

[94] R. Nieman , J. R. Carvalho , B. Jayee , et al., “Polyradical Character Assessment Using Multireference Calculations and Comparison With Density‐Functional Derived Fractional Occupation Number Weighted Density Analysis,” Physical Chemistry Chemical Physics 25 (2023): 27380–27393. 37792036 10.1039/d3cp03734g

[95] D. Rappoport and F. Furche , “Property‐Optimized Gaussian Basis Sets for Molecular Response Calculations,” Journal of Chemical Physics 133 (2010): 134105. 20942521 10.1063/1.3484283

[96] R. Ditchfield , “Molecular Orbital Theory of Magnetic Shielding and Magnetic Susceptibility,” Journal of Chemical Physics 56 (1972): 5688–5691.

[97] S. Gillhuber , Y. J. Franzke , and F. Weigend , “Paramagnetic NMR Shielding Tensors and Ring Currents: Efficient Implementation and Application to Heavy Element Compounds,” Journal of Physical Chemistry A 125 (2021): 9707–9723. 34723533 10.1021/acs.jpca.1c07793

[98] J. Juselius , R. Bast , H. Fliegl , et al., “The Gauge Including Magnetically Induced Current Density Method (GIMIC),” (2023).

[99] J. Ahrens , B. Geveci , and C. Law , The Visualization Handbook (Elsevier, 2005), 717.

[100] A. Karton , “A Computational Chemist's Guide to Accurate Thermochemistry for Organic Molecules,” WIREs Computational Molecular Science 6 (2016): 292, 10.1002/wcms.1249.

[101] W. Jiang , N. J. DeYonker , and A. K. Wilson , “Multireference Character for 3d Transition‐Metal‐Containing Molecules,” Journal of Chemical Theory and Computation 8 (2012): 460–468. 26596596 10.1021/ct2006852

[102] E. I. Ioannidis and H. J. Kulik , “Towards Quantifying the Role of Exact Exchange in Predictions of Transition Metal Complex Properties,” Journal of Chemical Physics 143 (2015): 034104. 26203011 10.1063/1.4926836

[103] A. Karton , “How Reliable Is DFT in Predicting Relative Energies of Polycyclic Aromatic Hydrocarbon Isomers? Comparison of Functionals From Different Rungs of Jacob's Ladder,” Journal of Computational Chemistry 38 (2017): 370, 10.1002/jcc.24669. 27859494

[104] S. H. Vosko , L. Wilk , and M. Nusair , “Accurate Spin‐Dependent Electron Liquid Correlation Energies for Local Spin Density Calculations: A Critical Analysis,” Canadian Journal of Physics 58 (1980): 1200–1211.

[105] Y. Zhao and D. G. Truhlar , “The M06 Suite of Density Functionals for Main Group Thermochemistry, Thermochemical Kinetics, Noncovalent Interactions, Excited States, and Transition Elements: Two New Functionals and Systematic Testing of Four M06‐Class Functionals and 12 Other Functionals,” Theoretical Chemistry Accounts 120 (2008): 215–241.

[106] J. Tao , J. P. Perdew , V. N. Staroverov , and G. E. Scuseria , “Climbing the Density Functional Ladder: Nonempirical Meta–Generalized Gradient Approximation Designed for Molecules and Solids,” Physical Review Letters 91 (2003): 146401. 14611541 10.1103/PhysRevLett.91.146401

[107] J. W. Furness , A. D. Kaplan , J. Ning , J. P. Perdew , and J. Sun , “Accurate and Numerically Efficient r2SCAN Meta‐Generalized Gradient Approximation,” Journal of Physical Chemistry Letters 11 (2020): 8208–8215. 32876454 10.1021/acs.jpclett.0c02405

[108] C. Lee , W. Yang , and R. G. Parr , “Development of the Colle‐Salvetti Correlation‐Energy Formula Into a Functional of the Electron Density,” Physical Review B 37 (1988): 785–789. 10.1103/physrevb.37.785 9944570

[109] A. D. Becke , “Density‐Functional Exchange‐Energy Approximation With Correct Asymptotic Behavior,” Physical Review A 38 (1988): 3098–3100. 10.1103/physreva.38.3098 9900728

[110] N. Mardirossian and M. Head‐Gordon , “Mapping the Genome of Meta‐Generalized Gradient Approximation Density Functionals: The Search for B97M‐V,” Journal of Chemical Physics 142 (2015): 074111. 25702006 10.1063/1.4907719

[111] Y. Zhang and W. Yang , “Comment on “Generalized Gradient Approximation Made Simple”,” Physical Review Letters 80 (1998): 890. 10.1103/PhysRevLett.77.3865 10062328

[112] J. Sun , A. Ruzsinszky , and J. Perdew , “Strongly Constrained and Appropriately Normed Semilocal Density Functional,” Physical Review Letters 115 (2015): 036402. 26230809 10.1103/PhysRevLett.115.036402

[113] B. Hammer , L. B. Hansen , and J. K. Nørskov , “Improved Adsorption Energetics Within Density‐Functional Theory Using Revised Perdew‐Burke‐Ernzerhof Functionals,” Physical Review B 59 (1999): 7413–7421.

[114] H. S. Yu , X. He , and D. G. Truhlar , “MN15‐L: A New Local Exchange‐Correlation Functional for Kohn–Sham Density Functional Theory With Broad Accuracy for Atoms, Molecules, and Solids,” Journal of Chemical Theory and Computation 12 (2016): 1280–1293. 26722866 10.1021/acs.jctc.5b01082

[115] R. Peverati and D. G. Truhlar , “M11‐L: A Local Density Functional That Provides Improved Accuracy for Electronic Structure Calculations in Chemistry and Physics,” Journal of Physical Chemistry Letters 3 (2012): 117–124. 10.1039/c2cp42025b 22910998

[116] V. N. Staroverov , G. E. Scuseria , J. Tao , and J. P. Perdew , “Comparative Assessment of a New Nonempirical Density Functional: Molecules and Hydrogen‐Bonded Complexes,” Journal of Chemical Physics 119 (2003): 12129–12137. 10.1063/1.4971853 28010100

[117] M. Bursch , H. Neugebauer , S. Ehlert , and S. Grimme , “Dispersion Corrected r2SCAN Based Global Hybrid Functionals: r2SCANh, r2SCAN0, and r2SCAN50,” Journal of Chemical Physics 156 (2022): 134105. 35395897 10.1063/5.0086040

[118] A. V. Krukau , O. A. Vydrov , A. F. Izmaylov , and G. E. Scuseria , “Influence of the Exchange Screening Parameter on the Performance of Screened Hybrid Functionals,” Journal of Chemical Physics 125 (2006): 224106. 17176133 10.1063/1.2404663

[119] Y. Zhao and D. G. Truhlar , “Design of Density Functionals That Are Broadly Accurate for Thermochemistry, Thermochemical Kinetics, and Nonbonded Interactions,” Journal of Physical Chemistry A 109 (2005): 5656–5667. 16833898 10.1021/jp050536c

[120] E. Brémond and C. Adamo , “Seeking for Parameter‐Free Double‐Hybrid Functionals: The PBE0‐DH Model,” Journal of Chemical Physics 135 (2011): 024106. 21766924 10.1063/1.3604569

[121] C. Holzer and Y. J. Franzke , “A Local Hybrid Exchange Functional Approximation From First Principles,” Journal of Chemical Physics 157 (2022): 034108. 35868924 10.1063/5.0100439

[122] M. Haasler , T. M. Maier , R. Grotjahn , S. Gückel , A. V. Arbuznikov , and M. Kaupp , “A Local Hybrid Functional With Wide Applicability and Good Balance Between (De)localization and Left–Right Correlation,” Journal of Chemical Theory and Computation 16 (2020): 5645–5657. 32697913 10.1021/acs.jctc.0c00498

[123] M. Casanova‐Páez and L. Goerigk , “Time‐Dependent Long‐Range‐Corrected Double‐Hybrid Density Functionals With Spin‐Component and Spin‐Opposite Scaling: A Comprehensive Analysis of Singlet–Singlet and Singlet–Triplet Excitation Energies,” Journal of Chemical Theory and Computation 17 (2021): 5165–5186. 34291643 10.1021/acs.jctc.1c00535

[124] H. S. Yu , X. He , S. L. Li , and D. G. Truhlar , “MN15: A Kohn–Sham Global‐Hybrid Exchange–Correlation Density Functional With Broad Accuracy for Multi‐Reference and Single‐Reference Systems and Noncovalent Interactions,” Chemical Science 7 (2016): 5032–5051. 30155154 10.1039/c6sc00705h PMC6018516

[125] L. Wittmann , H. Neugebauer , S. Grimme , and M. Bursch , “Dispersion‐Corrected r2SCAN Based Double‐Hybrid Functionals,” Journal of Chemical Physics 159 (2023): 224103. 38063220 10.1063/5.0174988

[126] L. Goerigk and S. Grimme , “Efficient and Accurate Double‐Hybrid‐Meta‐GGA Density Functionals—Evaluation With the Extended GMTKN30 Database for General Main Group Thermochemistry, Kinetics, and Noncovalent Interactions,” Journal of Chemical Theory and Computation 7 (2011): 291–309. 26596152 10.1021/ct100466k

[127] E. Brémond , J. C. Sancho‐García , A. J. Pérez‐Jiménez , and C. Adamo , “Communication: Double‐Hybrid Functionals From Adiabatic‐Connection: The QIDH Model,” Journal of Chemical Physics 141 (2014): 031101. 25053294 10.1063/1.4890314

[128] T. Yanai , D. P. Tew , and N. C. Handy , “A New Hybrid Exchange–Correlation Functional Using the Coulomb‐Attenuating Method (CAM‐B3LYP),” Chemical Physics Letters 393 (2004): 51–57.

[129] G. Santra , M. Cho , and J. M. L. Martin , “Exploring Avenues Beyond Revised DSD Functionals: I. Range Separation, With xDSD as a Special Case,” Journal of Physical Chemistry A 125 (2021): 4614–4627. 34009986 10.1021/acs.jpca.1c01294 PMC8279641

[130] Y.‐S. Lin , G.‐D. Li , S.‐P. Mao , and J.‐D. Chai , “Long‐Range Corrected Hybrid Density Functionals With Improved Dispersion Corrections,” Journal of Chemical Theory and Computation 9 (2013): 263–272. 26589028 10.1021/ct300715s

[131] S. Fürst , M. Kaupp , and A. Wodyński , “Range‐Separated Local Hybrid Functionals With Small Fractional‐Charge and Fractional‐Spin Errors: Escaping the Zero‐Sum Game of DFT Functionals,” Journal of Chemical Theory and Computation 19 (2023): 8639–8653. 37972297 10.1021/acs.jctc.3c00877

[132] Y. Tawada , T. Tsuneda , S. Yanagisawa , T. Yanai , and K. Hirao , “A Long‐Range‐Corrected Time‐Dependent Density Functional Theory,” Journal of Chemical Physics 120 (2004): 8425–8433. 15267767 10.1063/1.1688752

[133] S. Fürst , M. Haasler , R. Grotjahn , and M. Kaupp , “Full Implementation, Optimization, and Evaluation of a Range‐Separated Local Hybrid Functional With Wide Accuracy for Ground and Excited States,” Journal of Chemical Theory and Computation 19 (2023): 488–502. 10.1021/acs.jctc.2c00782 36625881

[134] A. Wodyński and M. Kaupp , ““Beyond‐Zero‐Sum” Range‐Separated Local Hybrid Functional With Improved Dynamical Correlation,” Journal of Chemical Theory and Computation 21 (2025): 7419–7429. 40679386 10.1021/acs.jctc.5c00699 PMC12355696

[135] E. Brémond , M. Savarese , A. J. Pérez‐Jiménez , J. C. Sancho‐García , and C. Adamo , “Range‐Separated Double‐Hybrid Functional From Nonempirical Constraints,” Journal of Chemical Theory and Computation 14 (2018): 4052–4062. 29923721 10.1021/acs.jctc.8b00261

[136] R. Peverati and D. G. Truhlar , “Improving the Accuracy of Hybrid Meta‐GGA Density Functionals by Range Separation,” Journal of Physical Chemistry Letters 2 (2011): 2810–2817.

[137] N. Mardirossian and M. Head‐Gordon , “Survival of the Most Transferable at the Top of Jacob's Ladder: Defining and Testing theωB97M(2) Double Hybrid Density Functional,” Journal of Chemical Physics 148 (2018): 241736. 29960332 10.1063/1.5025226 PMC5991970

[138] Y. Jin and R. J. Bartlett , “The QTP Family of Consistent Functionals and Potentials in Kohn‐Sham Density Functional Theory,” Journal of Chemical Physics 145 (2016): 034107. 27448874 10.1063/1.4955497

[139] N. Mardirossian and M. Head‐Gordon , “ωB97M‐V: A Combinatorially Optimized, Range‐Separated Hybrid, Meta‐GGA Density Functional With VV10 Nonlocal Correlation,” Journal of Chemical Physics 144 (2016): 214110. 27276948 10.1063/1.4952647

[140] N. Mardirossian and M. Head‐Gordon , “ωB97X‐V: A 10‐Parameter, Range‐Separated Hybrid, Generalized Gradient Approximation Density Functional With Nonlocal Correlation, Designed by a Survival‐Of‐The‐Fittest Strategy,” Physical Chemistry Chemical Physics 16 (2014): 9904–9924. 24430168 10.1039/c3cp54374a

[141] E. Brémond , A. J. Pérez‐Jiménez , J. C. Sancho‐García , and C. Adamo , “Range‐Separated Hybrid Density Functionals Made Simple,” Journal of Chemical Physics 150 (2019): 201102. 31153220 10.1063/1.5097164

[142] A. Najibi and L. Goerigk , “DFT‐D4counterparts of Leadingmeta‐Generalized‐Gradient Approximation and Hybrid Density Functionals for Energetics and Geometries,” Journal of Computational Chemistry 41 (2020): 2562–2572. 32870518 10.1002/jcc.26411

[143] H. Iikura , T. Tsuneda , T. Yanai , and K. Hirao , “A Long‐Range Correction Scheme for Generalized‐Gradient‐Approximation Exchange Functionals,” Journal of Chemical Physics 115 (2001): 3540–3544.

[144] Y. Zhao , N. González‐García , and D. G. Truhlar , “Benchmark Database of Barrier Heights for Heavy Atom Transfer, Nucleophilic Substitution, Association, and Unimolecular Reactions and Its Use to Test Theoretical,” Journal of Physical Chemistry A 109 (2005): 2012. 16833536 10.1021/jp045141s

[145] J. G. Brandenburg , C. Bannwarth , A. Hansen , and S. Grimme , “B97‐3c: A Revised Low‐Cost Variant of the B97‐D Density Functional Method,” Journal of Chemical Physics 148 (2018): 064104. 29448802 10.1063/1.5012601

[146] S. Grimme , A. Hansen , S. Ehlert , and J.‐M. Mewes , “r2SCAN‐3c: A “Swiss Army Knife” Composite Electronic‐Structure Method,” Journal of Chemical Physics 154 (2021): 064103. 33588555 10.1063/5.0040021

[147] J. G. Brandenburg , E. Caldeweyher , and S. Grimme , “Screened Exchange Hybrid Density Functional for Accurate and Efficient Structures and Interaction Energies,” Physical Chemistry Chemical Physics 18 (2016): 15519–15523. 27240749 10.1039/c6cp01697a

[148] M. Müller , A. Hansen , and S. Grimme , “ωB97X‐3c: A Composite Range‐Separated Hybrid DFT Method With a Molecule‐Optimized Polarized Valence Double‐ζ Basis Set,” Journal of Chemical Physics 158 (2023): 014103. 36610980 10.1063/5.0133026

[149] R. Sure and S. Grimme , “Corrected Small Basis Set Hartree‐Fock Method for Large Systems,” Journal of Computational Chemistry 34 (2013): 1672–1685. 23670872 10.1002/jcc.23317

[150] J. J. P. Stewart , “Optimization of Parameters for Semiempirical Methods V: Modification of NDDO Approximations and Application to 70 Elements,” Journal of Molecular Modeling 13 (2007): 1173–1213. 17828561 10.1007/s00894-007-0233-4 PMC2039871

[151] J. Řezáč , J. Fanfrlík , D. Salahub , and P. Hobza , “Semiempirical Quantum Chemical PM6 Method Augmented by Dispersion and H‐Bonding Correction Terms Reliably Describes Various Types of Noncovalent Complexes,” Journal of Chemical Theory and Computation 5 (2009): 1749–1760. 26610000 10.1021/ct9000922

[152] D. S. Levine , M. Shuaibi , E. W. C. Spotte‐Smith , et al., “The Open Molecules 2025 (OMol25) Dataset, Evaluations, and Models,” (2025), arXiv:2505.08762 [physics].

