# Supplementary material for: A Molecular “Thermometer” for Measuring Effective Non‐Local Exchange
Source: J Comput Chem. 2026 Jun 23;47(17):e70436. doi: 10.1002/jcc.70436 (PMC13288322; doi:10.1002/jcc.70436)
Supplement: Supplementary file 1 — Data S1: jcc70436‐sup‐0001‐Supinfo.pdf. [file JCC-47-0-s002.pdf]

Supporting Information: A molecular "thermometer" for measuring effective non-local exchange

Stefan Grimme,*+,[a] Marcel Müller,+,[a,b] Thomas Froitzheim,+,[a] Andreas Hansen,+,[a]

[a] Mulliken Center for Theoretical Chemistry, Clausius-Institute for Physical and Theoretical Chemistry, University of Bonn, Beringstraße 4, 53115 Bonn, Germany
E-mail: grimme@thch.uni-bonn.de

[b] Department of Chemistry, University of Toronto, 80 St. George St., Toronto, ON M5S 3H6, Canada; Vector Institute for Artificial Intelligence, W1140-108 College St., Schwartz Reisman Innovation Campus, Toronto, ON M5G 0C6, Canada

[+] These authors contributed equally.

1. Definition of NLX Measure

In the definition of the t_X measure, we use the following values for the HF and LDA reaction energies:

$$\Delta E_{\mathrm{HF}} = 66.4842118927492\,\mathrm{kcal\,mol^{-1}} \tag{1}$$

$$\Delta E_{\mathrm{LDA}} = -13.5873701146803\,\mathrm{kcal\,mol^{-1}} \tag{2}$$

2. ACID and MICD Plots

Figures 1 and 2 show the the combined anisotropy of the current-induced density (ACID) [1–3] and magnetically-induced current density (MICD) [4–6] at 0.5 and 2 Bohr above the molecular plane respectively. Both complement the plot for 1 Bohr above the molecular plane shown in Figure 1 of the main paper and are calculated at the same PBE0 [7]/def2-TZVPP [8,9] level of theory using gauge-including atomic orbitals. [10,11] The ACID at the consistently chosen isovalue remains fully connected throughout the π system of *carbo*-benzene, while a notable MICD remains even at a distance of 2 Bohr above the plane. Both are consistent with an aromatic ring-current also observed in previous studies of this and similar systems. [12–17] Meanwhile, the MICD in hexaethynylbenzene is substantially smaller and confined within a closer proximity to the molecular plane.

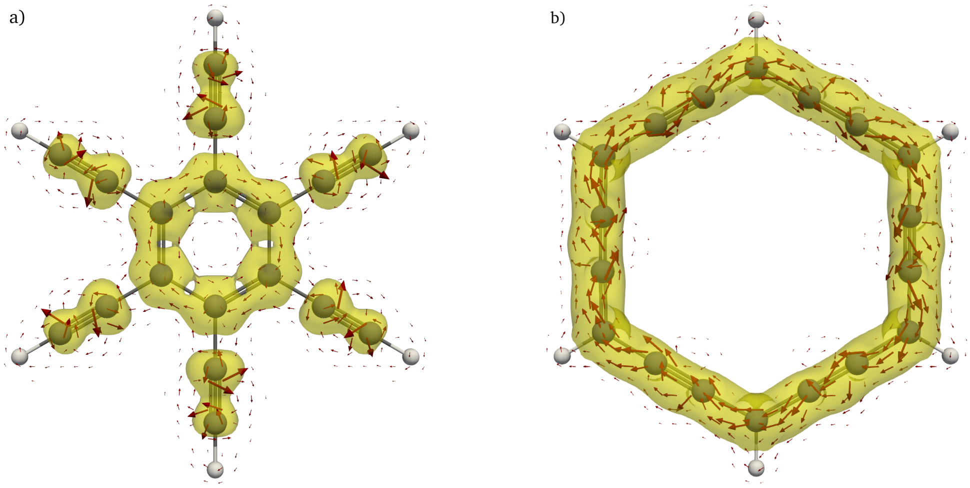

Figure 1. Combined scalar ACID (yellow, isovalue: 0.002) and vectorial MICD plot (red, selectively at 0.5 Bohr above the molecular plane vectors with a magnitude between 0.001 and 0.01, and scaled by a factor 5 for visibility) calculated at the PBE0/GIAO-def2-TZVPP level of theory for hexaethinylbenzene (a) and *carbo*-benzene (b). The erratic MICD vectors in (a) arise for grid points, which accidentally occur close to the carbon nuclei involved in the carbon-carbon triple bond.

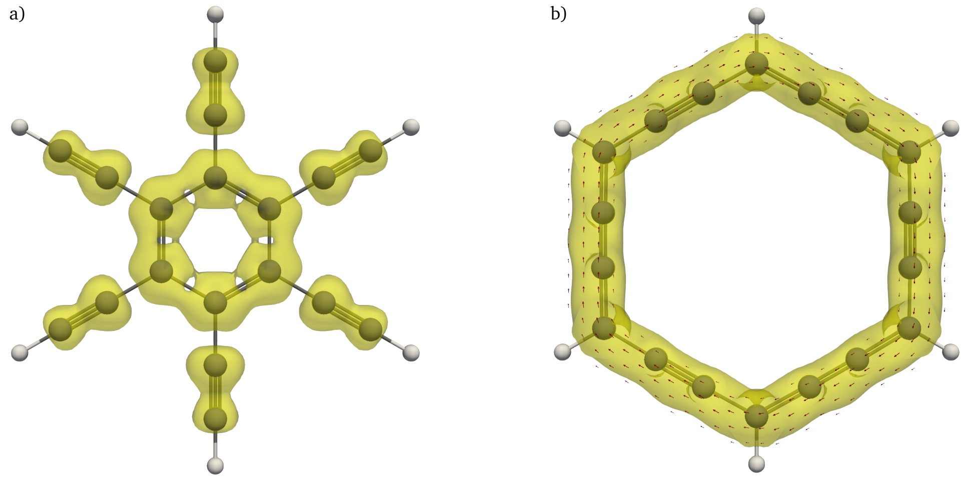

Figure 2. Combined scalar ACID (yellow, isovalue: 0.002) and vectorial MICD plot (red, selectively at 2 Bohr above the molecular plane vectors with a magnitude between 0.001 and 0.01, and scaled by a factor 5 for visibility) calculated at the PBE0/GIAO-def2-TZVPP level of theory for hexaethinylbenzene (a) and *carbo*-benzene (b).

To quantify the relative change in the ring-current during the bond rearrangement isomerization (BRI) from hexaethynylbenzene to *carbo*-benzene, we also integrated the MICD through the horizontal plane intersecting one C-C bond in the (expanded) benzene ring. The numerical integration was performed with the `GIMIC` program using a 200×200 grid with a twelfth-order Gauss integration in a rectangular plane spanning ± 14 Å in the z dimension and 0 to 10 Å in the x dimension. Additional tests were performed to confirm that the integrated magnetically-induced current (MIC) is converged to within 0.1 $\mathrm{nA\,T^{-1}}$ w.r.t. both the grid size and the extent of the integration plane. We performed the integration with a PBE-based hybrid functionals admixing between 0 and 100% Fock exchange.

The MIC in *carbo*-benzene is larger by about a factor of four than in hexaethynylbenzene (4.5 for $a_x = 0\%$ and 3.7 for $a_x = 100\%$), confirming the qualitative trends observed in the plotted MICD, Notably, the absolute value of the MIC changes only modestly with varying fractions of non-local Fock exchange (4.1 $\mathrm{nA\,T^{-1}}$ for *carbo*-benzene and 2.82 $\mathrm{nA\,T^{-1}}$ for hexaethynylbenzene), while the BRI energy varies by 71.6 $\mathrm{kcal\,mol^{-1}}$.

Table 1. Integrated magnetically-induced current (MIC) for carbobenzene and hexaethynylbenzene calculated with global hybrid (GH) PBE functionals employing between 0 and 100 % exact exchange. The integration was performed numerically using twelfth-order Gauss integration on a 200×200 point grid spanned from ± 14 Å in the z dimension and from 0 to 10 Å in the x dimension. The corresponding BRI reaction energy ΔE and the t_X non-local exchange (NLX) measure are tabulated (note that the values do not include a dispersion correction).

	carbo-benzene	hexaethynylbenzene		
a_X / %	MIC / $\mathrm{nA\,T^{-1}}$	MIC / $\mathrm{nA\,T^{-1}}$	ΔE / $\mathrm{kcal\,mol^{-1}}$	t_X
0	32.90	7.26	-17.17	-4.48
10	33.24	7.63	-10.70	3.61
20	33.87	7.98	-4.04	11.92
30	34.27	8.30	2.79	20.45
40	34.71	8.60	9.77	29.17
50	35.11	8.88	16.90	38.07
60	35.47	9.15	24.17	47.15
70	35.84	9.40	31.57	56.39
80	36.32	9.65	39.09	65.79
90	36.70	9.88	46.74	75.34
100	37.01	10.08	54.51	85.04

Given this small effect of the admixed fraction of Fock exchange on the MIC, the density functional approximation (DFA) error in the BRI seems to be predominantly functional-driven, following the distinction into density-driven and functional-driven errors of DFAs in Ref. 18. To further analyse the error source, we perform density-corrected density functional theory (DC-DFT) calculations,[18] again with the PBE-based hybrid functionals admixing between 0 and 100% Fock exchange. Aside from the Hartree-Fock (HF) wavefunction and density commonly employed in density-corrected (DC)-density functional theory (DFT), we also use the wavefunction and density calculated with the range-separated hybrid (RSH) ωB97M-V ($t_X = 62.8$), as a proxy for the correct density, similar to the approach in Ref. 19. Table 2 collects the resulting t_X values for the DC-DFT variants, together with the self-consistent results. t_X shows only minor DC effects, with the largest change being about ≈ 5 on the t_X-scale for PBE@HF relative to self-consistent PBE (PBE@ωB97M-V is even less affected). This confirms the conclusion from the MIC results that the BRI detects mainly functional-driven errors.

Table 2. BRI reaction energies ΔE and t_X NLX measures for self-consistent PBE hybrids, HF-density-corrected PBE hybrids, and ωB97M-V-density-corrected PBE hybrids employing between 0 and 100 % exact exchange. The values do not include a dispersion correction.

	self-consistent		@HF density		@ωB97M-V density	
a_X / %	ΔE / $\mathrm{kcal\,mol^{-1}}$	t_X	ΔE / $\mathrm{kcal\,mol^{-1}}$	t_X	ΔE / $\mathrm{kcal\,mol^{-1}}$	t_X
0	-17.17	-4.48	-21.74	-10.18	-19.46	-7.34
10	-10.70	3.61	-14.07	-0.60	-12.27	1.64
20	-4.04	11.92	-6.40	8.98	-5.08	10.62
30	2.79	20.45	1.27	18.56	2.11	19.60
40	9.77	29.17	8.94	28.14	9.29	28.58
50	16.90	38.07	16.61	37.72	16.48	37.56
60	24.17	47.15	24.28	47.30	23.67	46.53
70	31.57	56.39	31.95	56.87	30.86	55.51
80	39.09	65.79	39.62	66.45	38.05	64.49
90	46.74	75.34	47.29	76.03	45.24	73.47
100	54.51	85.04	54.96	85.61	52.43	82.45

3. FOD Diagnostic

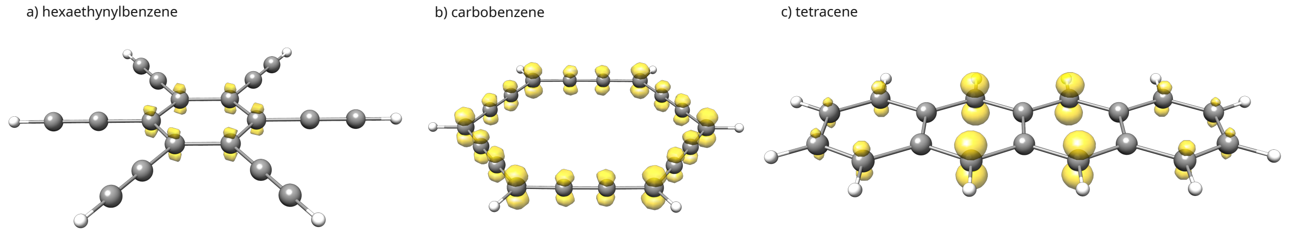

Figure 3. FOD plots (yellow, surface cut-off value: 0.005 e$^-$/Bohr^3) computed with RKS PBE0-D4/def2-TZVPPD (T_{el}=8830.5 K) using ORCA 6.0.1 for a) hexaethinylbenzene, b) *carbo*-benzene, and c) tetracene for comparison.

4. Coupled-Cluster Reference Values

Based on the multi-reference diagnostics performed, which yielded no signs of significant static correlation effects, the CCSD(T) method should be well-suited for calculating an accurate reference value for the reaction energy in question. To rule out basis set errors as much as possible, we decided to use an explicitly correlated, local CCSD(T) implementation (PNO-LCCSD(T)-F12b), which is applicable to these systems with very large basis sets and reproduces canonical CCSD(T) energies very well with tight threshold settings. The expansion of the basis set from aug-cc-pVQZ' to aug-cc-pV5Z', as well as the transition from tight to very tight threshold settings, has only a minimal effect (0.1 kcal) on the reaction energy. However, since we cannot completely rule out small amounts of post-(T) coupled-cluster (CC) contributions, we have estimated the error in our reference reaction energy to be slightly larger: 34.5 ± 0.25 kcal.

Table 3. BRI reaction energies ΔE (in kcal mol^{-1}) computed with PNO-LCCSD(T)-F12b as implemented in Molpro 2025.1. All calculations include a CABS singles correction.

Basis set	Threshold settings	ΔE / kcal mol^{-1}
**PNO-LCCSD(T)-F12b**		
aug-cc-pVQZ'	`TIGHT`	34.7
aug-cc-pVQZ'	`VTIGHT`	34.6
aug-cc-pV5Z'	`VTIGHT`	34.5

5. Basis Set and Geometry Optimization

The reaction energies ΔE with PBE0 range from 3.17 kcal mol^{-1} for def2-SVP [8], 5.17 kcal mol^{-1} for def2-TZVPP [8] and 5.44 kcal mol^{-1} for def2-QZVPPD [8,20]. Since the step from a triple-ζ to an augmented quadruple-ζ basis set is rather modest, we select the def2-TZVPP basis set to allow for a fast evaluation of the t_{X} metric for new DFAs.

To gauge the effect of the chosen geometry, we compare the used RI-MP2/def2-TZVPP geometries to reoptimized structures at the r^{2}SCAN-3c and ωB97X-3c level of theory. In both cases, we observe only a small RMSD in the atomic positions (hexaethynylbenzene: 0.011 Å or 0.023 Å and *carbo*-benzene: 0.017 Å or 0.035 Å for r^{2}SCAN-3c and ωB97X-3c, respectively), which has only a negligible effect on the calculated BRI energies. Notably, the admixture of Fock exchange in ωB97X-3c leads to a slight breaking of the D_{6h} symmetry in *carbo*-benzene due to localization of the alternatic double and triple bonds.

6. Correlation of BH76 mean deviations with the non-local exchange thermometer

Figure 4 compares the mean deviation (MD) for the BH76 benchmark set from the GMTKN55 collection [21,22] with the effective non-local exchange thermometer value t_{X} for the subset of functionals for which data were directly available. Where possible, dispersion-corrected GMTKN55 values were used, with D3(BJ) preferred over D3(0).

$$\mathrm{MD}_{\mathrm{BH76}} = 0.164\, t_{\mathrm{X}} - 8.454,$$

with $R^2 = 0.573$ and Pearson $r = 0.757$ for 46 matched functionals. As expected, larger t_X values generally reduce the strongly negative barrier-height bias typical of many (semi)local functionals.

However, the correlation is not universal. BLYP is the most pronounced exception: with $t_X = 9.22$, the fitted linear trend corresponds to an MD of $-6.94\,\text{kcal mol}^{-1}$, whereas the observed value is $+1.25\,\text{kcal mol}^{-1}$. Several Minnesota local and meta (m)-generalized-gradient approximation (GGA) functionals, in particular MN15-L, M11-L, and M06L, also lie well above the fitted line, indicating that their BH76 bias is shaped by parametrization and functional form beyond the effective non-local exchange measured by t_X. Excluding the three clearest outliers, BLYP, MN15-L, and M11-L, defined by internally studentized residuals with magnitude greater than 2, increases the Pearson correlation from $r = 0.757$ ($R^2 = 0.573$) to $r = 0.894$ ($R^2 = 0.800$); additionally excluding the visually separated M06L point gives $r = 0.926$ ($R^2 = 0.858$). Conversely, some conventional GGAs such as XLYP, BP86, RPBE, and mPWLYP remain more negative than expected from the global trend. At high t_X, the RSHs ωB97X-D3, M11, and ωB97X-V fall below the linear extrapolation, suggesting saturation or curvature in the high-exchange regime.

Overall, t_X captures a substantial systematic component of the BH76 MD, but the largest residuals show that it should be interpreted as a useful one-dimensional descriptor rather than a complete predictor of barrier-height performance.

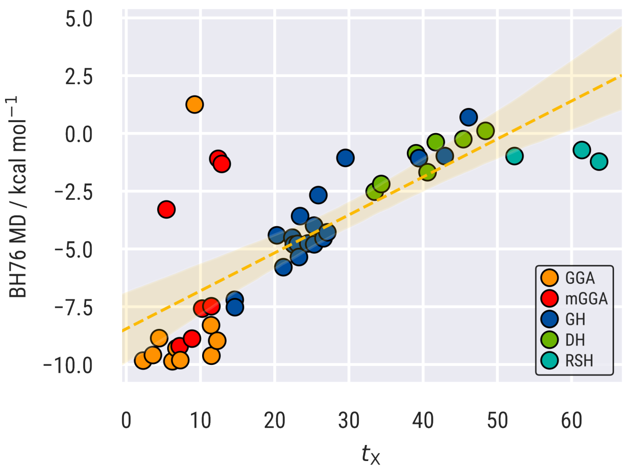

Figure 4. Correlation between the BH76 mean deviation (MD) and the effective non-local exchange thermometer value t_X. Points are colored by functional class. The dashed line shows a linear fit over the 46 matched functionals, yielding $R^2 = 0.573$ and Pearson $r = 0.757$.

Table 4. Functional class, non-local exchange measure t_X, and BH76 MD values in $kcal\,mol^{-1}$ for the functionals matched between the thermometer data and GMTKN55.

DFA	Type	t_X	BH76 MD / $kcal\,mol^{-1}$
PW91	GGA	2.28	-9.83
PBE	GGA	3.59	-9.59
rPW86PBE-D4	GGA	4.44	-8.86
M06L-D4	mGGA	5.42	-3.29
mPWLYP-D4	GGA	6.22	-9.86
mPWPW-D4	GGA	6.76	-9.30
TPSS-D4	mGGA	7.19	-9.21
BP86-D4	GGA	7.28	-9.81
revTPSS-D4	mGGA	8.86	-8.88
BLYP-D4	GGA	9.22	1.25
r^2SCAN-3c	mGGA	10.23	-7.58
revPBE-D4	GGA	11.42	-8.30
SCAN-D4	mGGA	11.47	-7.48
XLYP-D4	GGA	11.47	-9.62
RPBE-D4	GGA	12.26	-8.97
MN15-L-D3(0)	mGGA	12.40	-1.10
M11-L-D3(0)	mGGA	12.87	-1.32
TPSSh-D4	GH	14.62	-7.20
O3LYP-D4	GH	14.64	-7.52
HSE06-D4	GH	20.30	-4.41
B3P86-D4	GH	21.17	-5.79
PW1PW-D4	GH	22.38	-4.51
B3LYP-D4	GH	22.57	-4.81
B3PW91-D4	GH	23.10	-4.78
X3LYP-D4	GH	23.26	-5.35
PBE0-D4	GH	23.42	-3.58
mPW1LYP-D4	GH	24.52	-4.76
mPW1PW-D4	GH	25.30	-3.99
B1P86-D4	GH	25.32	-4.80
PW6B95-D4	GH	25.88	-2.67
TPSS0-D4	GH	26.58	-4.54
B1LYP-D4	GH	27.09	-4.28
M06-D4	GH	29.53	-1.06
B2PLYP-D4	DH	33.46	-2.52
mPW2PLYP-D4	DH	34.35	-2.19
B2GP-PLYP-D4	DH	39.05	-0.86
MN15-D3(BJ)	GH	39.43	-1.07
PWPB95-D4	DH	40.59	-1.68
DSD-BLYP-D3(BJ)	DH	41.70	-0.38
BMK-D3(BJ)	GH	42.92	-0.98
DSD-PBEP86-D3(BJ)	DH	45.41	-0.25
M06-2X-D3(0)	GH	46.14	0.70
DSD-PBEB95-D3(BJ)	DH	48.41	0.11
ωB97XD	RSH	52.31	-0.98
M11-D3(BJ)	RSH	61.38	-0.72
ωB97X-V	RSH	63.71	-1.22

7. Correlation of HOMO-LUMO energy gaps with the non-local exchange thermometer

Figure 5 correlates the t_X measure with the energy gap between the highest-occupied (HOMO) and lowest-unoccupied molecular orbitals (LUMO) for both hexaethynylbenzene (left) and *carbo*-benzene (right). The correlation is relatively strong with $R^2 = 0.885$ for hexaethynylbenzene and $R^2 = 0.757$ for *carbo*-benzene, respectively. However, certain details, such as the variance in t_X among GGA and mGGA functionals are not reproduced by the approximately constant HOMO-LUMO gaps. Since t_X is also more generally accessible for methods not based on a MOs, such as machine-learned interatomic potentials, we generally prefer the reaction energy-based t_X measure.

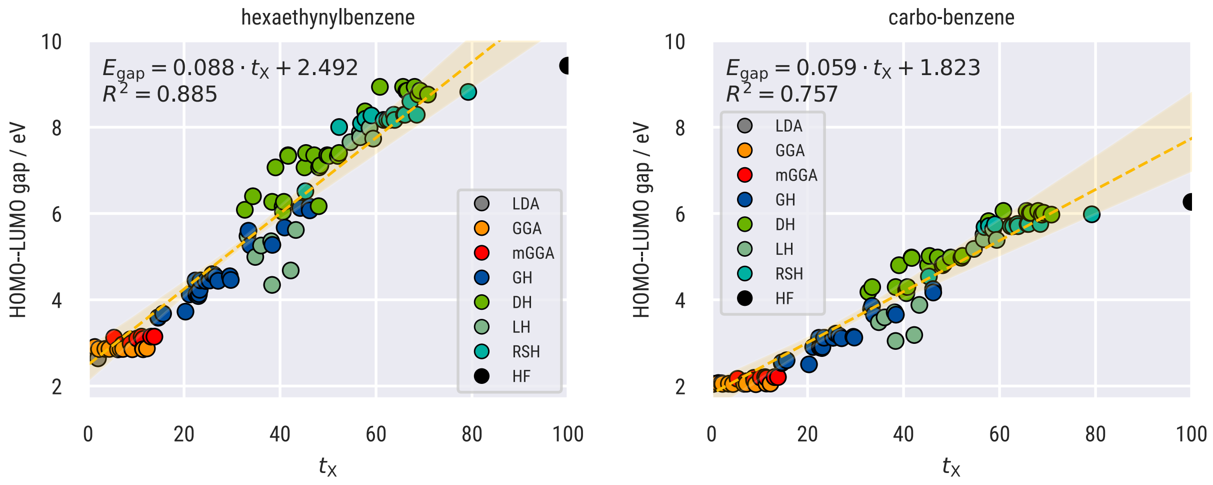

Figure 5. Correlation of the gap between the highest-occupied and lowest-unoccupied molecular orbitals and the effective non-local exchange thermometer value t_X for hexaethynylbenzene (left) and *carbo*-benzene (right). Points are colored by functional class. The dashed line shows a linear fit over the 101 matched functionals.

8. Additional Density Functionals and Dispersion Contributions

Table 5. Formal amount of Fock exchange a_X in %, reaction energies ΔE in kcal mol^{-1}, dispersion contributions to the reaction energy ΔE_{disp} in kcal mol^{-1}, and the NLX measure t_X. The methods are ordered based on the t_X measure. All DFT calculations employ the def2-TZVPP basis set and a functional-specific dispersion correction.

DFA	a_X	ΔE	ΔE_{disp}	t_X
LDA[23,24]	0	-13.6	–	0.0
PWP-D4[25,26]	0	-13.3	2.5	0.4
HFS[27]	0	-11.9	–	2.1
PW91-D4[28]	0	-11.8	5.3	2.3
PBE-D4[29]	0	-10.7	6.5	3.6
RPW86PBE-D4[30]	0	-10.0	7.2	4.4
M06L-D4[31]	0	-9.2	0.7	5.4
mPWLYP-D4[7]	0	-8.6	9.9	6.2
mPWPW-D4[7]	0	-8.2	9.8	6.8
TPSS-D4[32]	0	-7.8	8.7	7.2
BP86-D4[25,33]	0	-7.8	9.5	7.3
r^{2}SCAN-D4[34]	0	-6.5	1.9	8.8
revTPSS-D4[35]	0	-6.5	7.0	8.9
BLYP-D4[33,36]	0	-6.2	12.9	9.2
B97M-V[37]	0	-4.7	6.4	11.1
revPBE-D4[38]	0	-4.4	14.9	11.4
SCAN-D4[39]	0	-4.4	1.1	11.5
XLYP-D4[40]	0	-4.4	14.8	11.5
RPBE-D4[41]	0	-3.8	15.5	12.3
MN15-L-D3(0)[42]	0	-3.7	-0.0	12.4
M11-L-D3(0)[43]	0	-3.3	2.5	12.9
B97M-D4[44]	0	-3.1	8.0	13.1
B97M-D3(BJ)[44]	0	-2.5	8.6	13.8
TPSSh-D4[45]	10	-1.9	8.1	14.6
O3LYP-D4[46]	11.6	-1.9	9.8	14.6
r^{2}SCANh-D4[47]	10	-1.1	2.1	15.6
HSE06-D4[48]	25-0	2.7	5.5	20.3
B3P86-D4[49]	20	3.4	7.5	21.2
PW1PW-D4[50]	25	4.3	4.9	22.4
B3LYP-D4[51]	20	4.5	10.1	22.6
B97-D4[52]	19.4	4.8	10.2	23.0
B3PW91-D4[49]	20	4.9	10.1	23.1
X3LYP-D4[40]	21.8	5.0	9.1	23.3
PBE0-D4[50]	25	5.2	5.8	23.4
mPW1LYP-D4[7]	25	6.0	7.9	24.5
mPW1PW-D4[7]	25	6.7	7.9	25.3
B1P86-D4[25,33]	25	6.7	7.2	25.3
PW6B95-D4[53]	28	7.1	4.8	25.9
r^{2}SCAN0-D4[47]	25	7.4	2.3	26.1
TPSS0-D4[54]	25	7.7	7.7	26.6
B1LYP-D4[55]	25	8.1	10.4	27.1
M06-D4[31]	27	10.1	1.3	29.5
revPBE0-D4[56]	25	10.2	12.5	29.7
PBE0-DH[57]	50	12.6	–	32.7
TMHF-D4[58]	0-100	13	0.6	33.2
B2PLYP-D4[59]	53	13.2	4.8	33.5
PBE38-D4[22]	37.5	13.5	5.5	33.8
mPW2PLYP-D4[60]	53	13.9	3.1	34.4
LH20t-D4[61]	0-100	14.3	3.5	34.8
LH24x-D4[62]	0-100	15.3	2.9	36.0
LH24n-B95-D4[63]	0-100	17.0	6.4	38.1
PTPSSD3(0)[56]	50	17.1	2.1	38.3
CHYF-D4[64]	0-100	17.1	11.3	38.3
SCS-B2PLYP-D4[65][a]	53	17.1	4.8	38.3
revPBE38-D4[56]	37.5	17.2	10.5	38.4
B2GP-PLYP-D4[66]	65	17.7	3.1	39.0
MN15-D3(BJ)[67]	44	18.0	0.0	39.4
Pr^{2}SCAN50-D4[68]	50	18.9	1.7	40.5
PWPB95-D4[56]	50	18.9	3.4	40.6
SOS-B2PLYP-D4[65][a]	53	19.1	4.8	40.8
CF22D[69]	46.3	19.2	0.2	41.0
PBE-QIDH[70]	69	19.8	–	41.7
DSD-BLYP-D3(BJ)[71]	71	19.8	3.4	41.7
LH25nP-D4[72]	0-100	20.2	6.1	42.2
BMK-D3(BJ)[73]	42	20.8	7.3	42.9
Skala-D3[74]	0	20.9	8.9	43.1
LH24n-D4[63]	0-100	21.1	6.4	43.3
r^{2}SCAN50-D4[47]	50	21.8	2.5	44.2
SCS-B2GP-PLYP-D4[65][a]	65	22.5	3.1	45.1
CAM-B3LYP-D4[75]	19-65	22.7	6.1	45.3
DSD-PBEP86-D3(BJ)[71]	69	22.8	2.6	45.4
M06-2X-D3(0)[31]	54	23.4	0.1	46.1
BHHLYP-D4[49]	50	23.4	8.0	46.2
XYG3[76]	80.3	23.4	–	46.2
SCS-PBE-QIDH[65]	69	24.1	–	47.1
κPr^{2}SCAN50-D4[68]	50	24.9	1.7	48.0
SOS-B2GP-PLYP-D4[65][a]	65	24.9	3.1	48.1
DSD-PBEB95-D3(BJ)[71]	66	25.2	2.9	48.4
SOS-PBE-QIDH[65]	69	26.3	–	49.8
DM21-D3(BJ)[77]	0-100	26.6	8.9	50.2
revDSD-PBEP86-D4[78]	69	26.6	3.4	50.2
revDOD-PBEP86-D4[78]	69	28.1	3.8	52.0
Pr^{2}SCAN69-D4[68]	69	28.3	1.8	52.3
ωB97XD[79]	19.6-100	28.3	1.9	52.3
ωLH23tdP-D4[80]	0-100	30.2	1.9	54.7
ωLH23tdB-D4[80]	0-100	31.8	1.9	56.6
ωLH23tdE-D4[80]	0-100	31.8	1.9	56.7
ωr2SCAN-D4[68]	0-100	31.9	3.5	56.9
ωPr2SCAN50-D4[68]	50-100	32.6	2.0	57.7
LC-BLYP-D4[81]	0-100	32.7	0.8	57.8
ωLH23td-D4[80]	0-100	33.4	1.9	58.6
ωLH22t-D4[82]	0-100	33.4	1.9	58.6
ωB97X-D4[83]	15.8-100	33.7	1.0	59.0
ωLH25tdE-D4[84]	0-100	34.0	6.7	59.4
**CCSD(T) reference**	–	**34.5**	–	**60.0**
RSX-QIDH[85]	69-100	35.1	–	60.8
M11-D3(BJ)[86]	42.8-100	35.6	0.9	61.4
ωB97M-D3(BJ)[87]	15-100	35.7	5.5	61.6
ωB97M(2)[88]	62.2	36.0	2.1	61.9
CAM-QTP-01-D4[89]	23-100	36.3	1.9	62.3
ωB97M-D4[44]	15-100	36.3	6.1	62.3
ωB97M-V[90]	15-100	36.7	6.5	62.8
ωB97X-V[91]	16.7-100	37.4	6.4	63.7
ωB97M-D4rev[92]	15-100	37.5	7.4	63.9
SCS-RSX-QIDH[65]	69-100	39.0	–	65.6
ωB97X-D3(BJ)[87]	16.7-100	39.1	8.1	65.8
ωB97X-D4rev[92]	16.7-100	39.3	8.3	66.0
RSX-0DH[93]	50-100	39.5	–	66.3
SCS-ωB2GP-PLYP-D4[65][a]	65-100	39.7	3.1	66.5
ωB97-D4[83]	0-100	40.2	0.3	67.1
SOS-RSX-QIDH[65]	69-100	40.9	–	68.0
ωB97X-D4[44]	16.7-100	41.2	10.3	68.5
SCS-ωB2PLYP-D4[65][a]	53-100	41.5	4.8	68.8
SOS-ωB2GP-PLYP-D4[65][a]	65-100	41.8	3.1	69.2
SOS-ωB2PLYP-D4[65][a]	53-100	43.1	4.8	70.8
LC-PBE-D4[94]	0-100	49.8	7.2	79.2
HF-D4[95,96]	100	66.5	16.2	100.0

[a] DFT-D4 parameters adapted from the canonical double hybrid (DH).

Table 6. Formal amount of Fock exchange a_X in %, reaction energies ΔE in kcal mol^{-1}, dispersion contributions ΔE_{disp} in kcal mol^{-1}, and the NLX measure t_X for a set of composite DFT, SQM, and MLIP methods.

DFA/SQM/MLIP	a_X	ΔE	ΔE_{disp}	t_X
B97-3c [97]	0	-12.5	10.2	1.4
r^{2}SCAN-3c [98]	0	-5.4	2.1	10.2
PBEh-3c [99]	42	13.2	2.8	33.4
ωB97X-3c [92]	16.7-100	38.3	6.2	64.8
HF-3c [100]	100	97.3	12.1	138.5
GFN1-xTB [101]	0	14.0	2.9	34.5
GFN2-xTB [102]	0	9.8	5.4	29.2
g-xTB [103]	15-100	36.7	7.7	62.7
PM6-D3H4X [104,105]	100	44.0	–	72.0
AIMNet2 [106]	–	6.7	–	25.3
UMA-sm [107]	–	37.4	–	63.7

References

[1] R. Herges, D. Geuenich, *The Journal of Physical Chemistry A* **2001**, *105*, 3214.
[2] D. Geuenich, K. Hess, F. Köhler, R. Herges, *Chemical Reviews* **2005**, *105*, 3758.
[3] H. Fliegl, J. Jusélius, D. Sundholm, *The Journal of Physical Chemistry A* **2016**, *120*, 5658.
[4] J. Jusélius, D. Sundholm, J. Gauss, *The Journal of Chemical Physics* **2004**, *121*, 3952.
[5] D. Sundholm, H. Fliegl, R. J. Berger, *WIREs Computational Molecular Science* **2016**, *6*, 639.
[6] H. Fliegl, S. Taubert, O. Lehtonen, D. Sundholm, *Physical Chemistry Chemical Physics* **2011**, *13*, 20500.
[7] C. Adamo, V. Barone, *The Journal of Chemical Physics* **1998**, *108*, 664.
[8] F. Weigend, R. Ahlrichs, *Physical Chemistry Chemical Physics* **2005**, *7*, 3297.
[9] F. Weigend, *Physical Chemistry Chemical Physics* **2006**, *8*, 1057.
[10] F. London, *Journal de Physique et le Radium* **1937**, *8*, 397.
[11] R. Ditchfield, *The Journal of Chemical Physics* **1972**, *56*, 5688.
[12] C. Lepetit, C. Godard, R. Chauvin, *New Journal of Chemistry* **2001**, *25*, 572.
[13] C. Lepetit, B. Silvi, R. Chauvin, *The Journal of Physical Chemistry A* **2003**, *107*, 464.
[14] C. Zou, C. Lepetit, Y. Coppel, R. Chauvin, *Pure and Applied Chemistry* **2006**, *78*, 791.
[15] S. Jalife, M. Audiffred, R. Islas, S. Escalante, S. Pan, P. K. Chattaraj, G. Merino, *Chemical Physics Letters* **2014**, *610-611*, 209.
[16] K. Cocq, C. Lepetit, V. Maraval, R. Chauvin, *Chemical Society Reviews* **2015**, *44*, 6535.
[17] D. Arias-Olivares, A. Becerra-Buitrago, L. C. García-Sánchez, D. V. Moreno, R. Islas, *ACS Omega* **2024**, *9*, 10913.
[18] M.-C. Kim, E. Sim, K. Burke, *Physical Review Letters* **2013**, *111*, 073003.
[19] A. D. Kaplan, C. Shahi, P. Bhetwal, R. K. Sah, J. P. Perdew, *Journal of Chemical Theory and Computation* **2023**, *19*, 532.
[20] D. Rappoport, F. Furche, *The Journal of Chemical Physics* **2010**, *133*, 134105.
[21] Y. Zhao, N. González-García, D. G. Truhlar, *The Journal of Physical Chemistry A* **2005**, *109*, 2012.
[22] L. Goerigk, A. Hansen, C. Bauer, S. Ehrlich, A. Najibi, S. Grimme, *Physical Chemistry Chemical Physics* **2017**, *19*, 32184.
[23] P. a. M. Dirac, *Mathematical Proceedings of the Cambridge Philosophical Society* **1930**, *26*, 376.
[24] S. H. Vosko, L. Wilk, M. Nusair, *Canadian Journal of Physics* **1980**, *58*, 1200.
[25] J. P. Perdew, *Physical Review B* **1986**, *33*, 8822.
[26] J. P. Perdew, J. A. Chevary, S. H. Vosko, K. A. Jackson, M. R. Pederson, D. J. Singh, C. Fiolhais, *Physical Review B* **1992**, *46*, 6671.
[27] J. C. Slater, *Physical Review* **1951**, *81*, 385.
[28] Y. Wang, J. P. Perdew, *Physical Review B* **1991**, *44*, 13298.
[29] J. P. Perdew, M. Ernzerhof, K. Burke, *The Journal of Chemical Physics* **1996**, *105*, 9982.
[30] E. D. Murray, K. Lee, D. C. Langreth, *Journal of Chemical Theory and Computation* **2009**, *5*, 2754.
[31] Y. Zhao, D. G. Truhlar, *Theoretical Chemistry Accounts* **2008**, *120*, 215.
[32] J. Tao, J. P. Perdew, V. N. Staroverov, G. E. Scuseria, *Physical Review Letters* **2003**, *91*, 146401.
[33] A. D. Becke, *Physical Review A* **1988**, *38*, 3098.
[34] J. W. Furness, A. D. Kaplan, J. Ning, J. P. Perdew, J. Sun, *The Journal of Physical Chemistry Letters* **2020**, *11*, 8208.
[35] J. P. Perdew, A. Ruzsinszky, G. I. Csonka, L. A. Constantin, J. Sun, *Physical Review Letters* **2009**, *103*, 026403.
[36] C. Lee, W. Yang, R. G. Parr, *Phys. Rev. B* **1988**, *37*.
[37] N. Mardirossian, M. Head-Gordon, *The Journal of Chemical Physics* **2015**, *142*, 074111.
[38] Y. Zhang, W. Yang, *Physical Review Letters* **1998**, *80*, 890.
[39] J. Sun, A. Ruzsinszky, J. Perdew, *Physical Review Letters* **2015**, *115*, 036402.
[40] X. Xu, W. A. Goddard, *Proceedings of the National Academy of Sciences* **2004**, *101*, 2673.
[41] B. Hammer, L. B. Hansen, J. K. Nørskov, *Physical Review B* **1999**, *59*, 7413.
[42] H. S. Yu, X. He, D. G. Truhlar, *Journal of Chemical Theory and Computation* **2016**, *12*, 1280.
[43] R. Peverati, D. G. Truhlar, *The Journal of Physical Chemistry Letters* **2012**, *3*, 117.
[44] A. Najibi, L. Goerigk, *Journal of Computational Chemistry* **2020**, *41*, 2562.
[45] V. N. Staroverov, G. E. Scuseria, J. Tao, J. P. Perdew, *The Journal of Chemical Physics* **2003**, *119*, 12129.
[46] W.-M. Hoe, A. J. Cohen, N. C. Handy, *Chemical Physics Letters* **2001**, *341*, 319.
[47] M. Bursch, H. Neugebauer, S. Ehlert, S. Grimme, *The Journal of Chemical Physics* **2022**, *156*, 134105.
[48] A. V. Krukau, O. A. Vydrov, A. F. Izmaylov, G. E. Scuseria, *The Journal of Chemical Physics* **2006**, *125*, 224106.
[49] A. D. Becke, *The Journal of Chemical Physics* **1993**, *98*, 1372.

[50] C. Adamo, V. Barone, *The Journal of Chemical Physics* **1999**, *110*, 6158.
[51] P. J. Stephens, F. J. Devlin, C. F. Chabalowski, M. J. Frisch, *The Journal of Physical Chemistry* **1994**, *98*, 11623.
[52] A. D. Becke, *The Journal of Chemical Physics* **1997**, *107*, 8554.
[53] Y. Zhao, D. G. Truhlar, *The Journal of Physical Chemistry A* **2005**, *109*, 5656.
[54] S. Grimme, *The Journal of Physical Chemistry A* **2005**, *109*, 3067.
[55] C. Adamo, V. Barone, *Chemical Physics Letters* **1997**, *274*, 242.
[56] L. Goerigk, S. Grimme, *Journal of Chemical Theory and Computation* **2011**, *7*, 291.
[57] E. Brémond, C. Adamo, *The Journal of Chemical Physics* **2011**, *135*, 024106.
[58] C. Holzer, Y. J. Franzke, *The Journal of Chemical Physics* **2022**, *157*, 034108.
[59] S. Grimme, *J. Chem. Phys.* **2006**, *124*.
[60] T. Schwabe, S. Grimme, *Physical Chemistry Chemical Physics* **2006**, *8*, 4398.
[61] M. Haasler, T. M. Maier, R. Grotjahn, S. Gückel, A. V. Arbuznikov, M. Kaupp, *Journal of Chemical Theory and Computation* **2020**, *16*, 5645.
[62] A. V. Arbuznikov, A. Wodyski, M. Kaupp, *The Journal of Chemical Physics* **2024**, *161*, 164104.
[63] A. Wodyski, K. Glodny, M. Kaupp, *Journal of Chemical Theory and Computation* **2025**, *21*, 762.
[64] C. Holzer, Y. J. Franzke, *Journal of Chemical Theory and Computation* **2024**.
[65] M. Casanova-Páez, L. Goerigk, *Journal of Chemical Theory and Computation* **2021**, *17*, 5165.
[66] A. Karton, A. Tarnopolsky, J.-F. Lamère, G. C. Schatz, J. M. L. Martin, *The Journal of Physical Chemistry A* **2008**, *112*, 12868.
[67] H. S. Yu, X. He, S. L. Li, D. G. Truhlar, *Chemical Science* **2016**, *7*, 5032.
[68] L. Wittmann, H. Neugebauer, S. Grimme, M. Bursch, *The Journal of Chemical Physics* **2023**, *159*, 224103.
[69] Y. Liu, C. Zhang, Z. Liu, D. G. Truhlar, Y. Wang, X. He, *Nature Computational Science* **2023**, *3*, 48.
[70] E. Brémond, J. C. Sancho-García, A. J. Pérez-Jiménez, C. Adamo, *The Journal of Chemical Physics* **2014**, *141*, 031101.
[71] S. Kozuch, J. M. L. Martin, *Journal of Computational Chemistry* **2013**, *34*, 2327.
[72] A. Wodyski, M. Kaupp, *Journal of Computational Chemistry* **2026**, *47*, e70294.
[73] A. D. Boese, J. M. L. Martin, *The Journal of Chemical Physics* **2004**, *121*, 3405.
[74] G. Luise, C.-W. Huang, T. Vogels, D. P. Kooi, S. Ehlert, S. Lanius, K. J. H. Giesbertz, A. Karton, D. Gunceler, M. Stanley, W. P. Bruinsma, L. Huang, X. Wei, J. G. Torres, A. Katbashev, B. Máté, S.-O. Kaba, R. Sordillo, Y. Chen, D. B. Williams-Young, C. M. Bishop, J. Hermann, R. v. d. Berg, P. Gori-Giorgi, Accurate and scalable exchange-correlation with deep learning **2025**, arXiv:2506.14665 [physics] version: 1.
[75] T. Yanai, D. P. Tew, N. C. Handy, *Chemical Physics Letters* **2004**, *393*, 51.
[76] Y. Zhang, X. Xu, W. A. Goddard, *Proceedings of the National Academy of Sciences of the United States of America* **2009**, *106*, 4963.
[77] J. Kirkpatrick, B. McMorrow, D. H. P. Turban, A. L. Gaunt, J. S. Spencer, A. G. D. G. Matthews, A. Obika, L. Thiry, M. Fortunato, D. Pfau, L. R. Castellanos, S. Petersen, A. W. R. Nelson, P. Kohli, P. Mori-Sánchez, D. Hassabis, A. J. Cohen, *Science* **2021**, *374*, 1385.
[78] G. Santra, M. Cho, J. M. L. Martin, *The Journal of Physical Chemistry A* **2021**, *125*, 4614.
[79] Y.-S. Lin, G.-D. Li, S.-P. Mao, J.-D. Chai, *Journal of Chemical Theory and Computation* **2013**, *9*, 263.
[80] S. Fürst, M. Kaupp, A. Wodyski, *Journal of Chemical Theory and Computation* **2023**, *19*, 8639.
[81] Y. Tawada, T. Tsuneda, S. Yanagisawa, T. Yanai, K. Hirao, *The Journal of Chemical Physics* **2004**, *120*, 8425.
[82] S. Fürst, M. Haasler, R. Grotjahn, M. Kaupp, *Journal of Chemical Theory and Computation* **2023**, *19*, 488.
[83] J.-D. Chai, M. Head-Gordon, *The Journal of Chemical Physics* **2008**, *128*, 084106.
[84] A. Wodyski, M. Kaupp, *Journal of Chemical Theory and Computation* **2025**, *21*, 7419.
[85] E. Brémond, M. Savarese, A. J. Pérez-Jiménez, J. C. Sancho-García, C. Adamo, *Journal of Chemical Theory and Computation* **2018**, *14*, 4052.
[86] R. Peverati, D. G. Truhlar, *The Journal of Physical Chemistry Letters* **2011**, *2*, 2810.
[87] A. Najibi, L. Goerigk, *Journal of Chemical Theory and Computation* **2018**, *14*, 5725.
[88] N. Mardirossian, M. Head-Gordon, *The Journal of Chemical Physics* **2018**, *148*, 241736.
[89] Y. Jin, R. J. Bartlett, *The Journal of Chemical Physics* **2016**, *145*, 034107.
[90] N. Mardirossian, M. Head-Gordon, *The Journal of Chemical Physics* **2016**, *144*, 214110.
[91] N. Mardirossian, M. Head-Gordon, *Physical Chemistry Chemical Physics* **2014**, *16*, 9904.
[92] M. Müller, A. Hansen, S. Grimme, *The Journal of Chemical Physics* **2023**, *158*, 014103.
[93] E. Brémond, A. J. Pérez-Jiménez, J. C. Sancho-García, C. Adamo, *The Journal of Chemical Physics* **2019**, *150*, 201102.
[94] H. Iikura, T. Tsuneda, T. Yanai, K. Hirao, *The Journal of Chemical Physics* **2001**, *115*, 3540.
[95] D. R. Hartree, *Mathematical Proceedings of the Cambridge Philosophical Society* **1928**, *24*, 111.

[96] V. Fock, *Zeitschrift für Physik* **1930**, *61*, 126.
[97] J. G. Brandenburg, C. Bannwarth, A. Hansen, S. Grimme, *The Journal of Chemical Physics* **2018**, *148*, 064104.
[98] S. Grimme, A. Hansen, S. Ehlert, J.-M. Mewes, *The Journal of Chemical Physics* **2021**, *154*, 064103.
[99] J. G. Brandenburg, E. Caldeweyher, S. Grimme, *Physical Chemistry Chemical Physics* **2016**, *18*, 15519.
[100] R. Sure, S. Grimme, *Journal of Computational Chemistry* **2013**, *34*, 1672.
[101] S. Grimme, C. Bannwarth, P. Shushkov, *Journal of Chemical Theory and Computation* **2017**, *13*, 1989.
[102] C. Bannwarth, S. Ehlert, S. Grimme, *Journal of Chemical Theory and Computation* **2019**, *15*, 1652.
[103] T. Froitzheim, M. Müller, A. Hansen, S. Grimme, g-xTB: A General-Purpose Extended Tight-Binding Electronic Structure Method For the Elements H to Lr (Z=1103) **2025**.
[104] J. J. P. Stewart, *Journal of Molecular Modeling* **2007**, *13*, 1173.
[105] J. ezá, J. Fanfrlík, D. Salahub, P. Hobza, *Journal of Chemical Theory and Computation* **2009**, *5*, 1749.
[106] D. M. Anstine, R. Zubatyuk, O. Isayev, *Chemical Science* **2025**, *16*, 10228.
[107] B. M. Wood, M. Dzamba, X. Fu, M. Gao, M. Shuaibi, L. Barroso-Luque, K. Abdelmaqsoud, V. Gharakhanyan, J. R. Kitchin, D. S. Levine, K. Michel, A. Sriram, T. Cohen, A. Das, A. Rizvi, S. J. Sahoo, Z. W. Ulissi, C. L. Zitnick, UMA: A Family of Universal Models for Atoms **2025**, arXiv:2506.23971 [cs].
